# Unexpectedly High Propylene/Propane Separation Performance of Asymmetric Mixed-Matrix Membranes through Additive-Assisted *In Situ* ZIF-8 Filler Formation: Experimental and Computational Studies

**DOI:** 10.1021/acsami.3c19491

**Published:** 2024-03-14

**Authors:** Yinying Hua, Amro M. O. Mohamed, Gyeong Min Choi, Kie Yong Cho, Ioannis G. Economou, Hae-Kwon Jeong

**Affiliations:** ^†^Artie McFerrin Department of Chemical Engineering and ^‡^Department of Materials Science and Engineering, Texas A&M University, 3122 TAMU, College Station, Texas 77843-3122, United States; § Chemical Engineering Program, Texas A&M University at Qatar, PO Box 23874, Doha 23874, Qatar; ∥ Department of Industrial Chemistry, Pukyong National University, 45 Yongso-ro, Nam-gu, Busan 48513, Republic of Korea

**Keywords:** mixed-matrix membranes, metal−organic frameworks, zeolitic-imidazolate frameworks, gas separations, propylene/propane separation

## Abstract

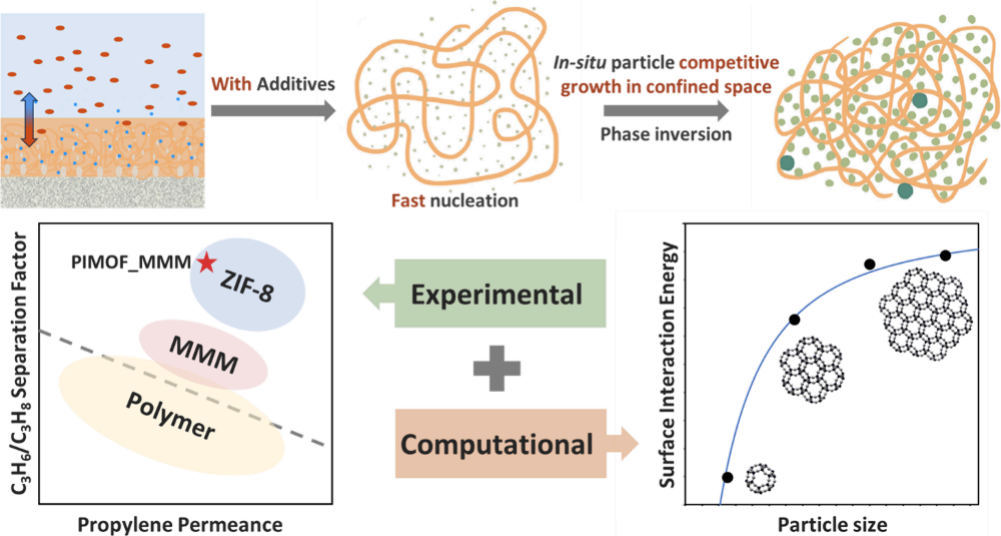

Zeolitic-imidazolate framework-8 (ZIF-8), composed of a zinc center tetrahedrally coordinated with 2-methylimidazolate linkers, has garnered extensive attention as a selective filler for propylene-selective mixed-matrix membranes (MMMs). Recently, we reported an innovative and scalable MMM fabrication approach, termed “phase-inversion in sync with *in situ* MOF formation” (PIMOF), aimed at addressing the prevailing challenges in MMM processing. In this study, we intend to investigate the effect of additives, specifically sodium formate and 1,4-butanediol, on the modification of ZIF-8 filler formation within the polymer matrix in order to further improve the separation performance of the asymmetric MMMs prepared by the PIMOF. Remarkably, MMMs prepared with sodium formate as an additive in the coagulation bath exhibited an unprecedented C_3_H_6_/C_3_H_8_ separation factor of 222.5 ± 1.8 with a C_3_H_6_ permeance of 10.1 ± 0.3 GPU, surpassing that of MMMs prepared without additives (a C3 separation factor of 57.7 ± 11.2 with a C3 permeance of 22.5 ± 4.5 GPU). Our computational work complements the experimental investigation by studying the effect of ZIF-8 nanoparticle size on the specific surface interaction energy and apertures of ZIF-8. Calculations indicate that by having smaller ZIF-8 nanoparticles, stronger interactions are present with the polymer affecting the aperture of ZIF-8 nanoparticles. This reduction in aperture size is expected to improve selectivity toward propylene by reducing the permeability of propylene. These results represent a significant advancement, surpassing the performance of all previously reported propylene-selective MMMs and most high-quality polycrystalline ZIF-8 membranes. The notably enhanced separation performance primarily arises from the formation of exceedingly small ZIF-8-like particles with an amorphous or poorly crystalline structure, corroborated by our computational work.

## Introduction

The separation of propylene (C_3_H_6_) from propane (C_3_H_8_, C3 separation, in short) constitutes one of the most energy-intensive processes, primarily attributed to the close resemblance in their physicochemical properties.^1,2^ Currently, C3 separation is carried out by thermally driven distillation processes. Despite the potential energy savings offered by membrane-based C3 separation,^3^ polymeric membranes hold sway in commercial gas separation markets due to their cost-effectiveness and scalability.^4^ However, these membranes suffer from what is known as the permeability/selectivity trade-off^5,6^ and plasticization at high pressure.^7^ While inorganic membranes exhibit remarkable thermal and chemical stability, coupled with high separation efficiency,^8^ their widespread adoption is hampered by prohibitively high costs resulting from the absence of cost-effective, robust, large-scale fabrication processes.^4^ In recent decades, mixed-matrix membranes (MMMs), comprising composites of polymers and inorganic fillers, have garnered significant research interest owing to their capacity to harness the strengths of both polymeric and inorganic membranes.^9^

Zeolitic-imidazolate framework-8 (ZIF-8), composed of zinc centers tetrahedrally coordinated with 2-methylimidazolate linkers, is one of the most promising membrane materials for propylene/propane separation.^10,11^ Notably, polycrystalline ZIF-8 membranes have demonstrated exceptional propylene/propane separation performance, achieving separation factors of approximately 300 with a propylene permeance of around 52 GPU.^12^ This outstanding propylene/propane separation performance of ZIF-8 results from its suitable effective aperture size (i.e., ca. 4.0–4.2 Å) due to the swing motion of the organic linker.^13^ On the other hand, ZIF-8 has been widely explored as a highly selective filler for propylene-selective MMMs as well.^14−16^

Despite the promises of MMMs, there are no commercial MMMs available for gas separations. This is mainly due to the considerable challenges of producing asymmetric MMMs in either hollow fiber or flat sheet forms by the conventional blending methods.^17^ Recently, our group has reported two novel *in situ* MMM processing methods, the first-generation polymer-modification-enabled *in situ* MOF formation (PMMOF)^8,18^ and second-generation phase-inversion in sync with MOF formation (PIMOF).^19^ The PMMOF, based on the chemical modification of polyimide, not only facilitated the *in situ* formation of asymmetric MMMs but also led to the creation of the first MMM hollow fiber modules.^20^ On the other hand, the second-generation PIMOF process combines the polymer phase inversion and in situ ZIF-8 formation without necessitating chemical modification of the polymer, where a zinc-ion-containing polymer film undergoes non-solvent-induced phase inversion in a linker-containing coagulation bath while ZIF-8 particles form *in situ* inside the polymer film by counter diffusion of zinc ions and linkers. High-performance asymmetric MMMs were rapidly formed in a scalable manner, and the membranes showed unexpectedly high C_3_H_6_/C_3_H_8_ separation performances (C_3_H_6_/C_3_H_8_ separation factor of ca. 106.9 and C_3_H_6_ permeance of ca. 7.5 GPU).^19^ Though impressive, further enhancements are highly desirable to augment the recovery and purity of propylene through membrane-based C3 separation processes.

The separation performance of MMMs is significantly influenced by the microstructure of the filler particles, encompassing factors such as shape, size, and external surface termination.^21−23^ A viable approach to exert control over the microstructure of ZIF-8 and other MOFs involves coordination modulation through the incorporation of auxiliary monodentate ligands.^24−26^ The modulating ligands with different chemical functionalities can act as competitive ligands in coordination equilibria and/or as bases in deprotonation equilibria, thereby modulating coordination and consequently forming ZIF-8 with different size and shapes.^27^ For instance, sodium formate has proven to be an effective additive in the fabrication of ZIF-8 polycrystalline membranes. This is attributed to its dual role in not only facilitating the heterogeneous nucleation of ZIF-8 crystals but also encouraging the intergrowth of these crystals. Shah et al.^28^ corroborated that the presence of sodium formate promoted the intergrowth of ZIF-8 crystals, resulting in the production of high-quality membranes. On the other hand, 1,4-butanediol was used as an additive to facilitate the deprotonation of 2-methylimidazolate as well as to promote preferential growth, resulting in improved microstructure of ZIF-8 membranes.^29^ Aside from modulating ligands, the confined growth of ZIFs can also be employed to tailor their microstructure and, occasionally, their crystal structures. This strategy offers substantial advantages for customizing their properties and thus expanding their applications. Li et al.^30^ demonstrated controlled ZIF-8 single-crystal growth within a three-dimensionally ordered macroporous polystyrene replica. Through precise adjustment of the precursor concentration, they were able to finely tailor the growth patterns and morphologies. More interestingly, Ma et al.^31^ showed that an amorphous ZIF-8-like structure can be formed in a confined γ-alumina mesoporous layer, leading to enhanced gas separation performance.

Based on the above observations, we hypothesized that the incorporation of additives into the PIMOF process could influence the coordination dynamics of linkers with zinc ions within the polymer matrix, subsequently impacting the formation of ZIF-8 fillers within the polymer and thereby altering their microstructures. Given the unique characteristics of the PIMOF process, the rapid solidification of the polymer restricts the growth of ZIF-8 particles would also affect the microstructure and/or crystal structure of fillers, potentially affecting the C3 separation performance of the membranes. To explore this hypothesis, we systematically investigated two additives, namely sodium formate and 1,4-butanediol, by incorporating them into the coagulation bath. We conducted a comprehensive analysis of the effects of these additives on the microstructure and performance of MMMs. MMMs by the PIMOF with additives were characterized, tested, and compared with other reported propylene-selective membranes, including those MMMs by the PIMOF without additives.

Our experimental investigation was further supported here through detailed Molecular Dynamics (MD) simulations based on a realistic representation of the MMMs using developed coarse-grained (CG) models. The simulations were used to examine the interfacial interaction between the filler and polyimide employing different ZIF nanoparticle sizes. Therefore, the simulations examined the effects of filler particle size, induced defects, and loading ratio of the filler on structural and compatibility properties of the ZIF and polymer.

## Experimental Section

### Materials

4,4-(Hexafluoroisopropylidene) diphthalic anhydride 2,4,6-trimethyl-1,3-phenylenediamine (6FDA-DAM) (*M*_w_ 148k, PDI 2.14) was purchased from Akron Polymer Systems Inc. 1-Methyl-2-pyrrolidone (NMP, C_5_H_9_NO, >99.0%, Sigma-Aldrich), tetrahydrofuran (THF, C_4_H_8_O, >99.0%, Alfa Aesar), methanol (MeOH, CH_3_OH, >99.8%, VWR International), ethanol (EtOH, C_2_H_5_OH, 94–96%, Alfa Aesar), sodium formate (NaCOOH, >99.0%, BioUltra, Sigma-Aldrich), 1,4-butanediol (HO(CH_2_)_4_OH, 99%, Sigma-Aldrich), zinc nitrate hexahydrate (ZnN, Zn(NO_3_)_2_·6H_2_O, 98%, Sigma-Aldrich), and 2-methylimidazole (HmIm, C_4_H_6_N_2_, 99%, Sigma-Aldrich) were used. All chemicals were used as received without further purification.

### Fabrication of MMMs with Additives

Membranes were prepared by following our recently reported procedure.^19^ A polymer solution was prepared by dissolving 15 wt % of 6FDA-DAM polyimide (hereafter, PI) in 40.7 w% NMP and 20.3 wt % THF, followed by the addition of nonsolvent additives of 6 wt % ZnN and 18 wt % EtOH. The solution was shaken overnight on a lab shaker at room temperature until homogeneous. The homogeneous polymer solution was then poured on a polytetrafluoroethylene (PTFE) filter (Advantec, T080A090C) with a casting thickness of ca. 340 μm for membranes with 1,4-butanediol and ca. 270 μm with sodium formate at room temperature. The casted polymer film was immersed into an aqueous coagulation bath containing 1,4-butanediol (hereafter, BD) or sodium formate (hereafter, SF) in 150 mL of DI water. The additive concentration varied from 0.2 to 1.0 M, and the samples are named “PIMOF_*x*BD” and “PIMOF_*y*SF”, respectively, where *x* and *y* represent additive molar concentration. For example, PIMOF_0.2BD is a membrane prepared in a coagulation bath with 0.2 M of 1,4 butanediol as an additive. The HmIm concentration was fixed at 2 M in the coagulation bath for all the membranes. The resulting membrane sample was kept in the coagulation bath for 1 h and transferred to a MeOH solution for solvent exchange for 1 h. The sample was then slowly dried under an environment nearly saturated with MeOH for 48 h. The resulting dried membrane was stored in a Petri dish for characterization and testing.

### Characterizations

Scanning electron microscope (SEM) images were taken at an acceleration voltage of 5 keV and a working distance of 15 mm using a JEOL JSM-7500F instrument. Transmission electron microscopy (TEM) analysis was conducted using an FEI Tecnai G2 F20 Super-Twin FE-TEM instrument operating at 120 kV. TEM samples were prepared using a Tescan LYRA-3 Model GMH dual-beam FIB instrument. X-ray diffraction (XRD) patterns were collected using a Miniflex II diffractometer (Rigaku) with Cu Kα radiation (λ = 1.5406 Å) in the 2θ range of 5–40°. A Nicolet iS5 spectrophotometer equipped with iD7 ATR (Thermo Scientific) was used to obtain attenuated total reflectance Fourier transform infrared (ATR-FTIR) spectra at a resolution of 5 cm^–1^ with 16 scans in the span of 4000–400 cm^–1^. Thermogravimetric analysis (TGA) was performed using a Q50 apparatus (TA Instruments) in the temperature range of 25–800 °C at the heating rate of 10 °C min^–1^ under an air flow of 60 cm^3^ min^–1^. N_2_ adsorption isotherms were taken using an ASAP 2020 plus instrument (Micromeritics) at 77 K.

### Gas Permeation Measurements

Equimolar binary propylene/propane separation performances of the membranes were determined using the Wicke–Kallenbach technique at room temperature under atmospheric pressure. A feed gas mixture was provided with a flow rate of 100 cm^3^ min^–1^. Argon sweeping gas flowed to the permeate side with the same flow rate as the feed gas. A membrane was placed in a custom-made permeation cell and sealed with rubber rings. The composition of the permeate was detected by a gas chromatograph (GC 7890A, Agilent) equipped with a flame ionization detector (FID) and an HP-plot Q column.

### Computational Simulation Methodology

#### Coarse-Grained Force Fields

**Figure 1:**
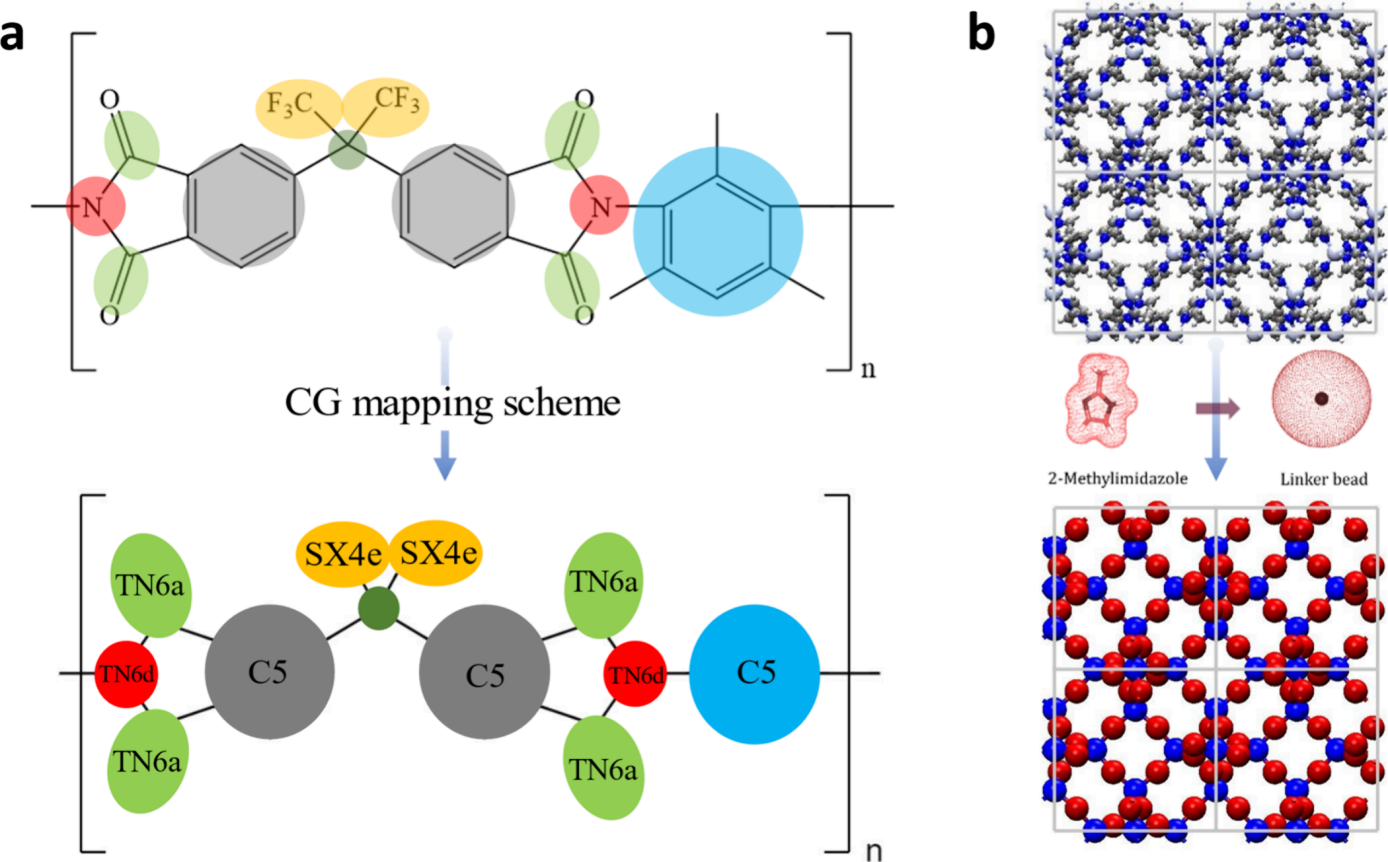
Coarse-grained model mapping for (a) 6FDA-DAM polymer and (b) ZIF-8. The polymer force field results in reducing the number of interaction sites of a 6FDA-DAM monomer from 83 to 12, whereas the ZIF-8 force field reduces the interaction sites to only 2.

Our computational endeavor focuses on simulating nanoparticles of variable sizes in MMM to elucidate the structural characteristics and affinity within polymeric frameworks. We recently developed an efficient CG modeling approach for both ZIF-8^32^ and 6FDA-DAM^33^ polymer that provides an accurate representation of the physical properties of the materials with reduced computational effort compared to fully atomistic representation. Figure 1 illustrates a schematic representation of the CG models, emphasizing the reduction in interaction sites for (a) 6FDA-DAM and (b) ZIF-8, a crucial step for facilitating simulations of large systems in mixed matrix membrane (MMM) form. The CG models employed in our study have undergone development and validation for several properties. Comprehensive details regarding the simulation methodologies and the corresponding results of these CG models are reported in the Supporting Information. For the polymer 6FDA-DAM, the targeted properties in the force field development encompass structure (e.g., radius of gyration and end-to-end distance), free volume, packing density, glass transition temperature, and gas adsorption. In parallel, the force field for the MOF is meticulously designed to yield accurate structural parameters, thermodynamic profiles of gas adsorption, and insights into amorphicity under pressurization. A pivotal aspect of this study is the investigation of amorphous-induced pressurization, and in particular, its role in the potential amorphization occurring as ZIF particles form *in situ* within the 6FDA-DAM medium. This focus is instrumental in shedding light on the morphological transformations as a result of pressurization that may transpire during the formation of ZIF particles, thereby contributing significantly to our understanding of the interplay between MOFs and polymeric structures in MMM systems.

#### Surface Interaction Energy and Structural Indicators

Utilizing crystallographic data, ZIF-8 nanoparticles of varying dimensions were reconstructed using the CG approach, deriving their crystalline nature from atomistic simulations. This process entailed the use of equilateral facets characterized by Miller indices ⟨1,0,0⟩, ⟨−1,0,0⟩, ⟨0,1,0⟩, ⟨0,–1,0⟩, ⟨0,0,1⟩, and ⟨0,–1,0⟩, each exhibiting equal binding energies. The resultant crystal morphology was a product of the Bravais–Friedel–Donnay–Harker (BFDH) theoretical crystal morphology algorithm, adeptly implemented within the Mercury software framework.^34^ In the creation of these nanoparticles, the NanoCrystal tool was employed, with specific Miller indices being integral to their design. Concurrently, a MMM system was conceptualized using 6FDA-DAM polymer chains, each comprising a trimer structure. A notable aspect of this system was the incorporation of smaller nanoparticles, maintaining a consistent surface termination ratio (Zn:L = 1:3), thereby enhancing surface intermolecular interactions.

For the simulation and equilibration of the MMM system, an annealing process was pivotal given the nature of the glassy polyimide. This process not only facilitated the local energy minimization of the glassy polymer but also allowed for structural reconfiguration in response to varying weight fractions in a later study. The simulations start under standard ambient conditions in the NpT ensemble, utilizing the GROMACS platform. This phase was succeeded by a gradual temperature elevation to 1000 K and reduction to 300 K.

For the postsimulation analysis, an analytical approach was adopted. This included single-point energy calculations to evaluate interaction energies and adsorption energies of individual polymer chains. A focal point of this analysis was the quantification of surface interaction energy between the MOF and polymer within the MMM, using the expressions
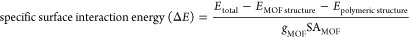
1
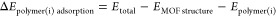
2where *E*_total_, *E*_MOF structure_, *E*_polymeric structure_, and *E*_polymer(i)_ are the energies of the total MMM system, ZIF-8 nanoparticle, 6FDA-DAM polymeric system and individual polymer chain, respectively. *g*_MOF_ is the mass of the nanoparticle, and SA_MOF_ is the external surface of the nanoparticle.

Further, the external surface area of select nanoparticles (SA_MOF_) was estimated through a combination of cluster analysis and size distribution assessments, facilitated by the MAPS software.^35^ This software employs a machine learning algorithm, Density-Based Spatial Clustering of Applications with Noise (DBSCAN), and Monte Carlo methods for precise volume and surface area calculations. Our findings, illustrated in Figure S11a, underscore the suitability of a spherical model for nanoparticles in the 2–4 nm range, with a gradual shift to a cubic representation at 7 nm. Notably, the 2 nm particle exhibited an external area smaller than that of a perfect sphere, attributable to its rhombic dodecahedral structure, as detailed in Figure S11b in the Supporting Information.

## Results and Discussion

**Figure 2:**
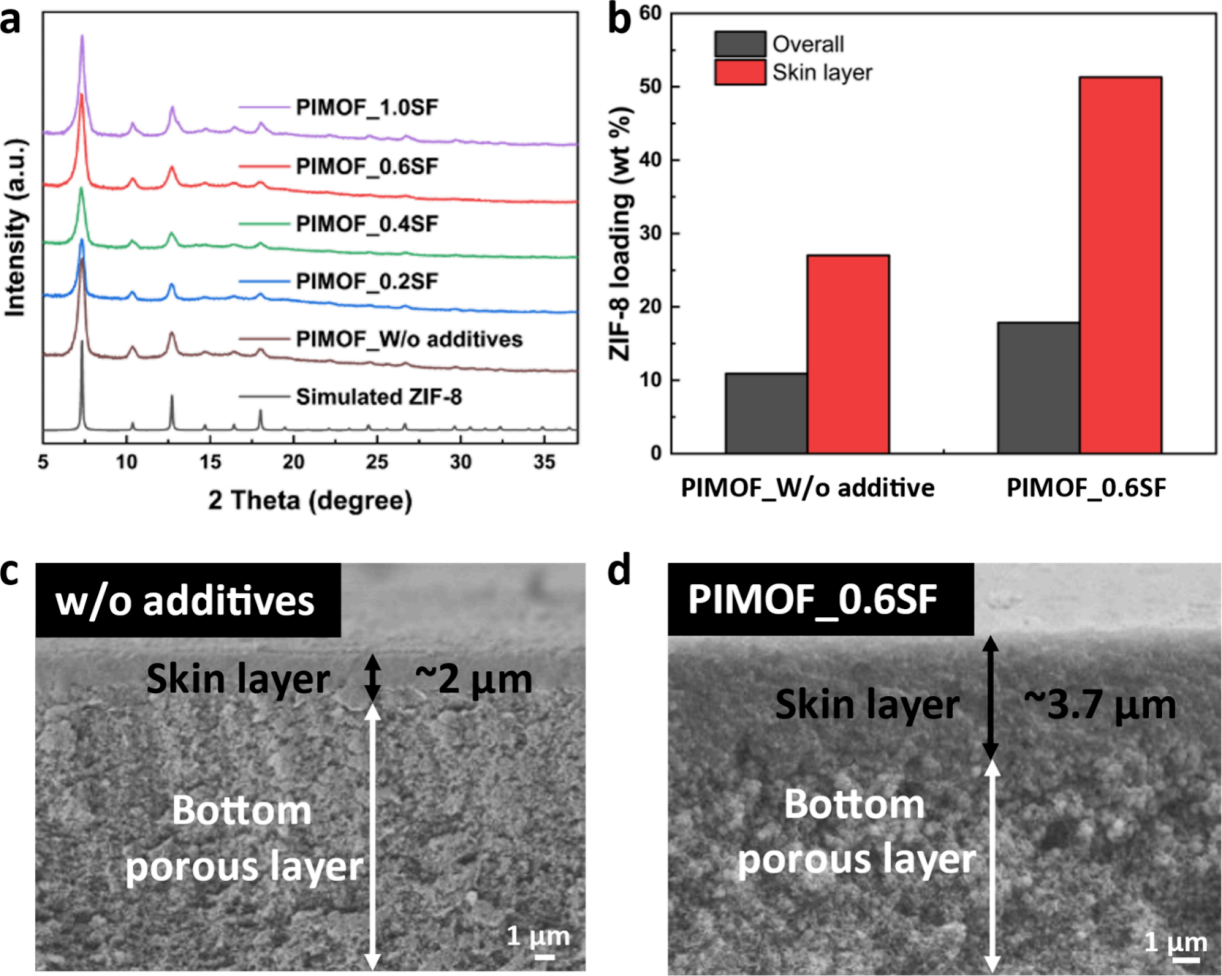
(a) XRD patterns of MMMs fabricated without additives and with sodium formate in the coagulation bath. (b) ZIF-8 loading percentages in the skin layer and all through the membranes. (c) Cross-section SEM images of PIMOF_w/o additives and (d) PIMOF_0.6SF.

Figure 2a presents the XRD patterns of the MMMs prepared using the PIMOF method, showcasing varying concentrations of sodium formate in the coagulation bath. The XRD patterns confirm the *in situ* formation of ZIF-8 inside the polymer matrices for all membranes. The overall ZIF-8 loading percentage in the PIMOF_0.6SF membrane is ca. 17.9 wt % as determined by TGA (Figure 2b). It is worth noting that this overall loading percentage encompasses the filler particles formed in both the skin layer and the bottom layer, as indicated in Figure 2c,d. As presented in our previous work,^19^ we derived estimates for the average ZIF-8 loadings in the upper skin layers (ca. 3 μm) by combining TGA with EDS elemental analysis (Figure 2b, Figures S1 and S2, and Table S1). Briefly, the overall ZIF-8 loadings were obtained from the TGA analysis. The average ZIF-8 loading in the skin layer of the PIMOF_0.6SF was then estimated by multiplying the overall ZIF-8 loading by the ratio of the average zinc counts in the top ca. 3 μm layers to those spanning the entire cross-section of the membrane, as determined by the EDS line scan. The PIMOF_0.6SF shows the overall ZIF-8 loading and the average ZIF-8 loading in the skin layer at 17.9 and 51.3 wt %, respectively, which are ca. 64.2% and ca. 89.9% higher than that of the PIMOF without additives, respectively.

Figure 2c,d and Figure S3 show scanning electron microscopy (SEM) images of the cross sections of the MMMs. Irrespective of the additive used, all membranes exhibit asymmetric microstructures, characterized by distinct dense layers on top of porous bottom layers. The thickness of the apparent skin layer in the membrane without additives is estimated to be ca. 2 μm. In contrast, the membrane prepared in the presence of sodium formate displays a much thicker skin layer, measured to be ca. 3.7 μm. The observed augmentation in the thickness of the skin layer is likely owing to the delayed demixing process during phase inversion.^36^ The inclusion of additives in the coagulation bath diminishes the activity of the nonsolvent as well as its rate of exchange with the solvent, thereby delaying the demixing process.

**Figure 3:**
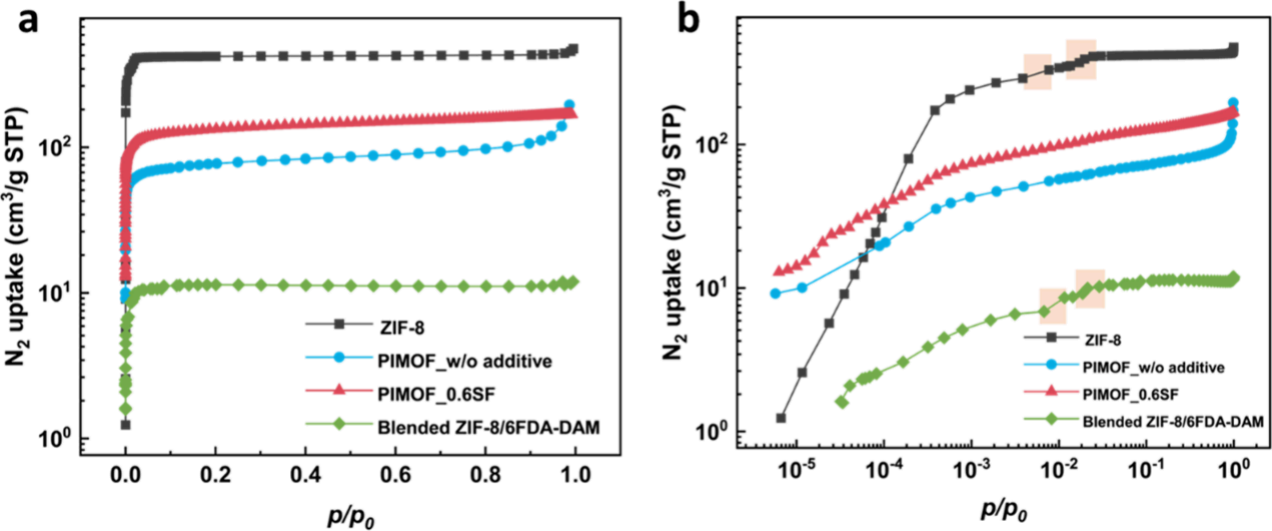
N_2_ adsorption isotherm at 77 K of a PIMOF_0.6SF in comparison with those of ZIF-8, PIMOF_w/o additive MMM, and conventional blended ZIF-8/6FDA-DAM MMM (20 wt %): (a) linear-log scale and (b) log–log scale.

Figure 3 displays the N_2_ adsorption isotherm of a PIMOF_0.6SF MMM alongside the isotherms of ZIF-8 powder, ZIF-8/6FDA-DAM MMM prepared by the conventional blending method, and PIMOF_w/o additive MMM. As depicted in Figure 3a, both PIMOF_MMMs exhibit smaller pore volumes than ZIF-8 powder, primarily due to the presence of the nonporous polymer phase of the PIMOF_MMMs. Interestingly, PIMOF_0.6SF shows a notably greater pore volume than PIMOF_MMM without the additive, likely due to its higher overall ZIF-8 loading. As can be seen in the log–log scale isotherms (see Figure 3b), both ZIF-8 powder and the ZIF-8-containing MMM prepared by a conventional physical blending method display well-known two-step gate-opening phenomena resulting from the linker swing motion (see the highlighted regions in Figure 3b).^37^ On the contrary, PIMOF_MMMs clearly do not exhibit gate-opening steps. The absence of gate-opening steps strongly suggests the stronger interaction between the filler surface and polymer, restricting the linker swing and consequently the molecular sieving properties of the filler particles.^38^

**Figure 4:**
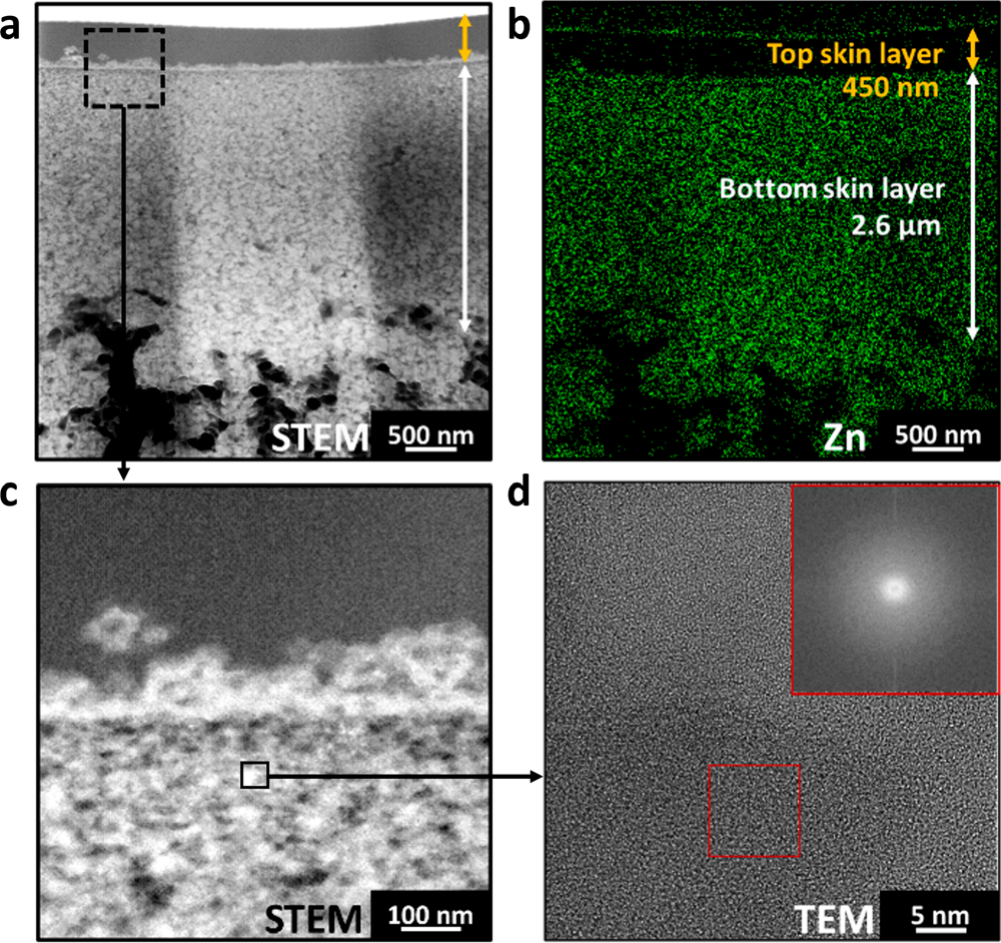
(a) STEM image of the cross-section of PIMOF_0.6SF. (b) Zinc EDS elemental mapping image of the same sample as shown in (a). (c) STEM image of the interface between the top skin layer and bottom skin layer. (d) HRTEM of the potential ZIF-8 particles and the corresponding FFT pattern (inset).

**Figure 5:**
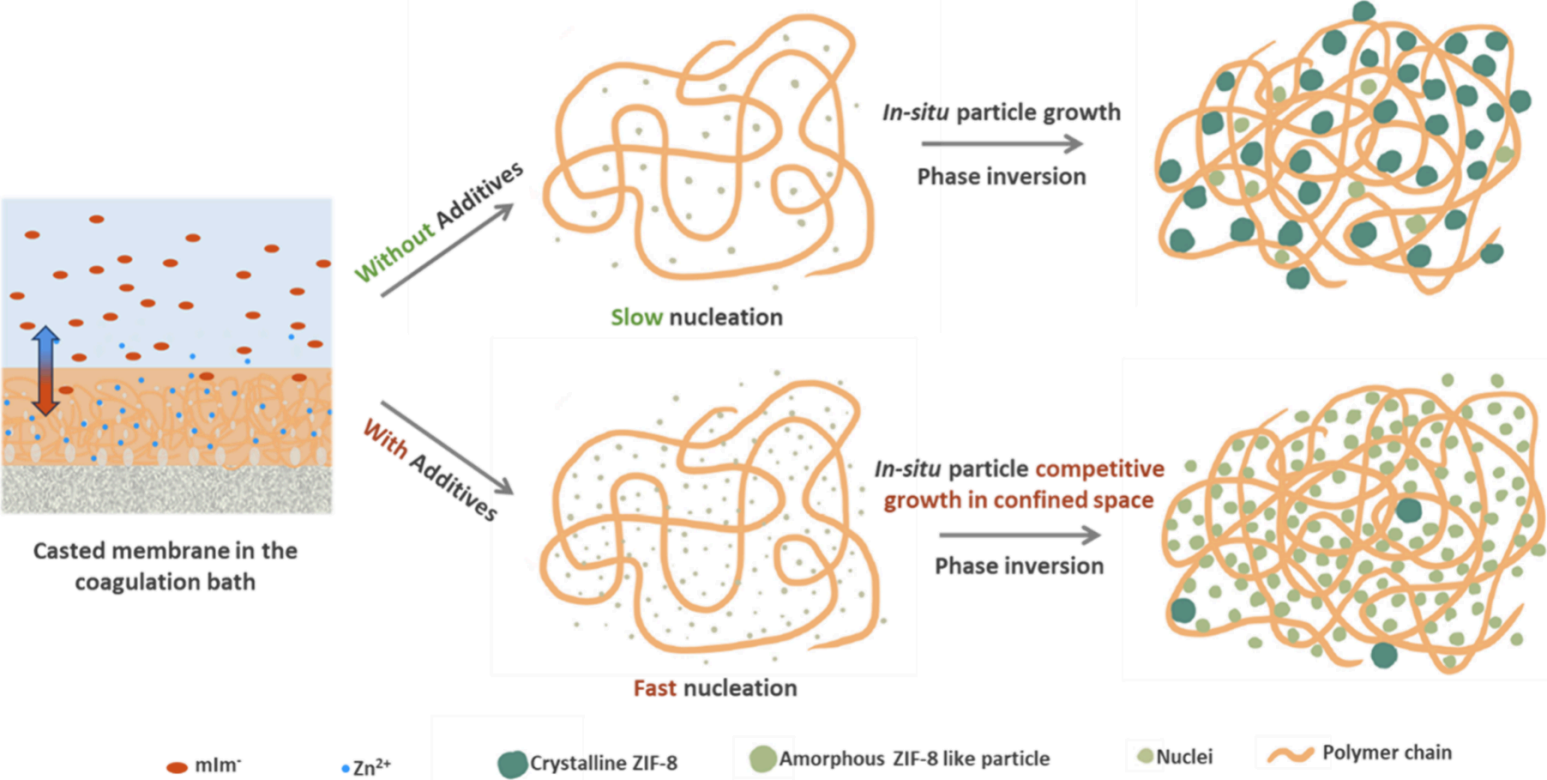
Illustration of PIMOF_Additives MMMs with faster nucleation compared to those without additives and the formation of ZIF-8 like particles within the confined space.

To examine the microstructure of the membranes, transmission electron microscopy (TEM) and scanning transmission electron microscopy (STEM) analyses were conducted on the PIMOF_0.6SF membrane. Figure 4a displays the STEM image of the top 4 μm thick apparent skin layer of PIMOF_0.6SF, revealing a two-layer structure: a top skin dense layer with a thickness of approximately 450 nm and a bottom skin layer measuring around 2.6 μm in thickness. The STEM-EDS mapping in Figure 4b shows that the top skin layer appears dense with a sparse zinc signal, indicating it is made of mostly polymer. A similar observation of a dense polymer layer was reported earlier,^19^ and its formation was attributed to zinc ions leaching from the polymer film’s top surface during solvent exchange before ZIF-8 formation. Figure 4c presents a STEM image of an enlarged region from an area in the vicinity of the top and bottom skin layers. Numerous bright spots can be observed in the image, which are presumed to be coalesced zinc compounds. Unfortunately, it was not possible to obtain high-resolution TEM (HRTEM) images to reveal the crystalline structure of these zinc compounds (see Figure 4d) due to their instability under the electron beam,^31,39^ strongly suggesting that these zinc compounds are either amorphous or poorly crystalline phases. In stark contrast with the case without additives, numerous crystalline ZIF-8 nanoparticles of ca. 5–10 nm were easily found in HRTEM images.^19^ In general, the formation of ZIF-8 crystals in a solution consists of three stages: nucleation, growth, and stationary.^40,41^ At the initial stage of nucleation, there exist amorphous or metastable nuclei,^40^ followed by their growth into crystals. The nucleation and growth of ZIF-8 are known to be dependent strongly on the concentration of deprotonated HmIm (i.e., mIm) in the growth solution.^26^ It is surmised that sodium formate acts as a deprotonator (i.e., nucleation promoter) rather than a competitive ligand,^42^ thereby resulting in a relatively high concentration of deprotonated linkers (i.e., higher mIm/HmIm) in the coagulation bath as compared to that without sodium formate. As more deprotonated linkers diffuse into a zinc-containing polymer film that simultaneously undergoes phase inversion, more nuclei form rapidly inside the confined spaces of polymers, quickly depleting HmIm ligands necessary for the nuclei to grow into a crystalline phase, thereby leading to the formation of amorphous or poorly crystalline ZIF-8-like particles (see Figure 5). In fact, Tsapatsis and co-workers^31^ made a similar observation that when vapor-phase grown inside the mesopores (2–5 nm) of a γ-alumina layer, an amorphous ZIF-8-like phase was formed and they attributed the formation of the amorphous ZIF-8-like phase to its confinement growth within the minuscule pores of the γ-alumina layer.

**Figure 6:**
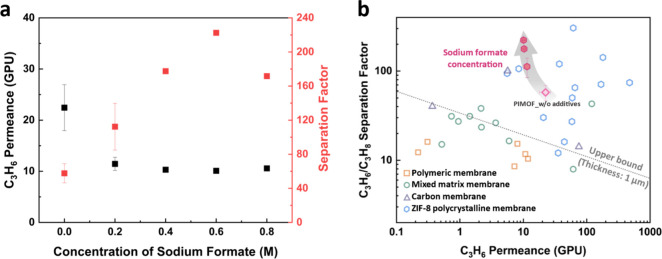
(a) The C3 separation performance of the PIMOF_MMMs with various concentrations of sodium formate in the coagulation bath. (b) The C3 separation performances of the PIMOF_MMMs with additives in comparison with other membranes.

To investigate the impact of the additive on the gas separation performance of the membranes, binary propylene/propane separation tests were conducted at room temperature. The propylene permeance and propylene/propane selectivity are plotted as a function of additive concentration (Figure 6a and Table S2). Figure 6b presents the binary propylene/propane (C3) separation performances of the PIMOF_MMMs, as compared to other membranes, including conventionally prepared ZIF-containing MMMs and polycrystalline ZIF-8 membranes.^12,31,43−51^ As the additive concentration in the coagulation bath increases, the separation factor increases and then ramps down at 0.8 M of sodium formate while the propylene permeance decreases and reaches a plateau. With 0.6 M sodium formate in the coagulation bath, the separation factor reaches up to ca. 222.5 ± 1.8, which is higher than those for most of the high-performance ZIF-8 membranes. The decreased permeance results possibly from the increased skin layer thickness (ca. 2 μm of PIMOF_w/o additives vs ca. 3.7 μm of PIMOF_0.6SF). The enhanced C3 gas separation performance of PIMOF_0.6SF can be attributed to (1) increased filler loading and (2) more restricted linker swing motion, reducing the effective aperture of the ZIF-8-like fillers as compared to the PIMOF_MMMs without additives.^19^ Due to the accelerated nucleation, a large number of small amorphous/poorly crystalline ZIF-8-like particles form in the skin layers of the PIMOF_SF MMMs. These amorphous/poorly crystalline ZIF-8-like particles are expected to have higher surface energy than their crystalline counterparts of the PIMOF_MMMs without additives, thereby rendering stronger interaction with the polymer and consequently restricting linker swing motion further, ultimately further reducing their effective aperture sizes and enhancing C3 separation performances. Meanwhile, amorphous or partially crystalline ZIFs could possibly bring higher C3 separation performances than their crystalline counterparts possibly due to their improved gas transport and uptake capacities.^52,53^

As presented in Figure 2d, there are crystalline ZIF-8 particles formed in the bottom porous layer of the PIMOF_0.6SF. In order to confirm that instead of these crystalline ZIF-8 particles in the bottom porous layer, the ZIF-8-like particles in the skin layer are responsible for the separation performance, selective etching of crystalline ZIF-8 particles of the PIMOF_0.6SF MMM was attempted by washing in a weak acid solution (0.1 M acetic acid). Due to the dense polymer top skin layer and surrounding polymer, it was surmised that the ZIF-8-like particles in the skin layer could be partially protected from acid attack, while the crystalline ZIF-8 particles in the highly porous bottom layer could be easily accessed by acid. After immersing the MMM in 200 mL of acid solution for 60 s, followed by washing with DI water, there was a significant reduction in the intensity of the characteristic (110) peak of ZIF-8 (Figure S5) due to the substantial removal of crystalline ZIF-8. Though decreased, the membrane after the acid treatment for 60 s still exhibits a relatively high separation factor of ca. 65.2 with increased C3 permeance of ca. 17.9 GPU. The observed decrease in separation factor is plausibly due to the destructive acid-washing process. Nevertheless, this observation strongly suggests that the ZIF-8-like particles formed in the skin layer are instrumental in the observed separation performance of the PIMOF_0.6SF MMMs.

**Figure 7:**
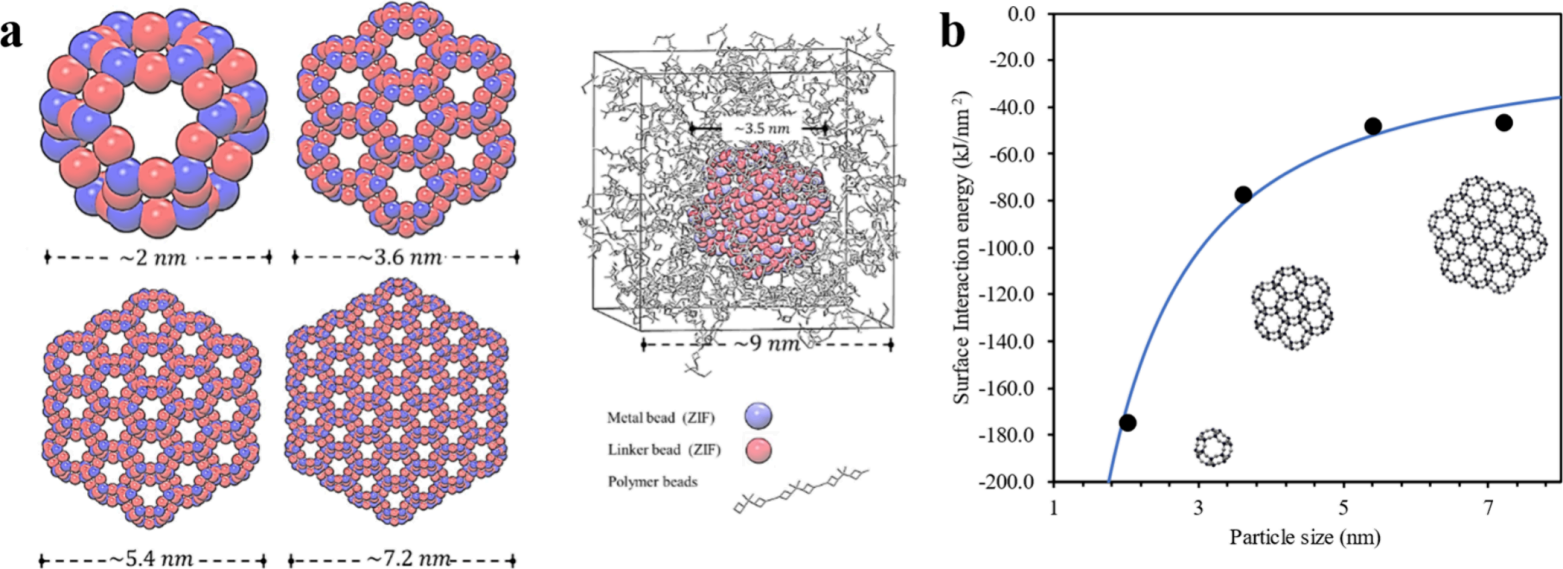
(a) Representation of MMM using coarse-grained simulations with different ZIF-8 nanoparticle sizes. (b) The surface interaction energy of MMM using different ZIF-8 nanoparticle sizes at 10 wt % loading.

In order to understand the interaction between the polymer chain and the ZIF-8 nanoparticles, CG simulations were performed. ZIF-8 nanoparticles, of the same termination (Zn:L = 1:3), with sizes of 2, 3.6, 5.4, and 7.2 nm were inserted, using Monte Carlo methods, in the 6FDA-DAM matrix (see Figure 7a). It is important to note that sizes are a product of constraining the composition and external surface connectivity inherent to the ZIF-8 topology. The analysis focused on the surface interaction energy between the MOF and polymer within the MMM as depicted in Figure 7b. A notable observation was the significant enhancement of surface interaction energy with decreasing nanoparticle size, indicated by the negative values signifying prevalent attractive forces between the polymer matrix and MOF nanoparticles. The observed correlation aligns with the findings from the N_2_ adsorption analysis (i.e., absence of the gate-opening steps). The ZIF-8 particles, synthesized through the PIMOF process within the size range of <5 nm, exhibit notable affinity with the polymer chain. Particularly noteworthy is the discernible trend wherein ZIF-8 like fillers synthesized with sodium formate not only manifest a reduced size but also demonstrate an enhanced level of interaction. It is also observed that a form of strain or distortion in the ZIF-8 crystal which indicates the interaction of the ZIF-8 nanoparticle surface with the polymer matrix may potentially affect the crystallinity of ZIF-8, especially at the interface. Our study revealed a decrease in nanoparticle mobility with size reduction, as evidenced by diffusion coefficient analysis. Despite challenges in accurately representing diffusivities using Mean Square Displacement (MSD) in this system, the marked difference in mobility, as detailed in Table S12, confirms higher compatibility and enhanced dispersion of nanoparticles, potentially leading to improved separation performance.

To validate the magnitude of interactions, we analyzed 2 nm nanoparticles (10 wt %) in MMM assessing individual polymer chains to plot interaction energy profiles against nanoparticle structure. Figure S12 highlights these interactions; our findings, especially for chains near nanoparticle surfaces (∼−150 kJ/mol), align closely with existing literature on adhesion energies in functionalized MOF-based MMMs.^54^

**Figure 8:**
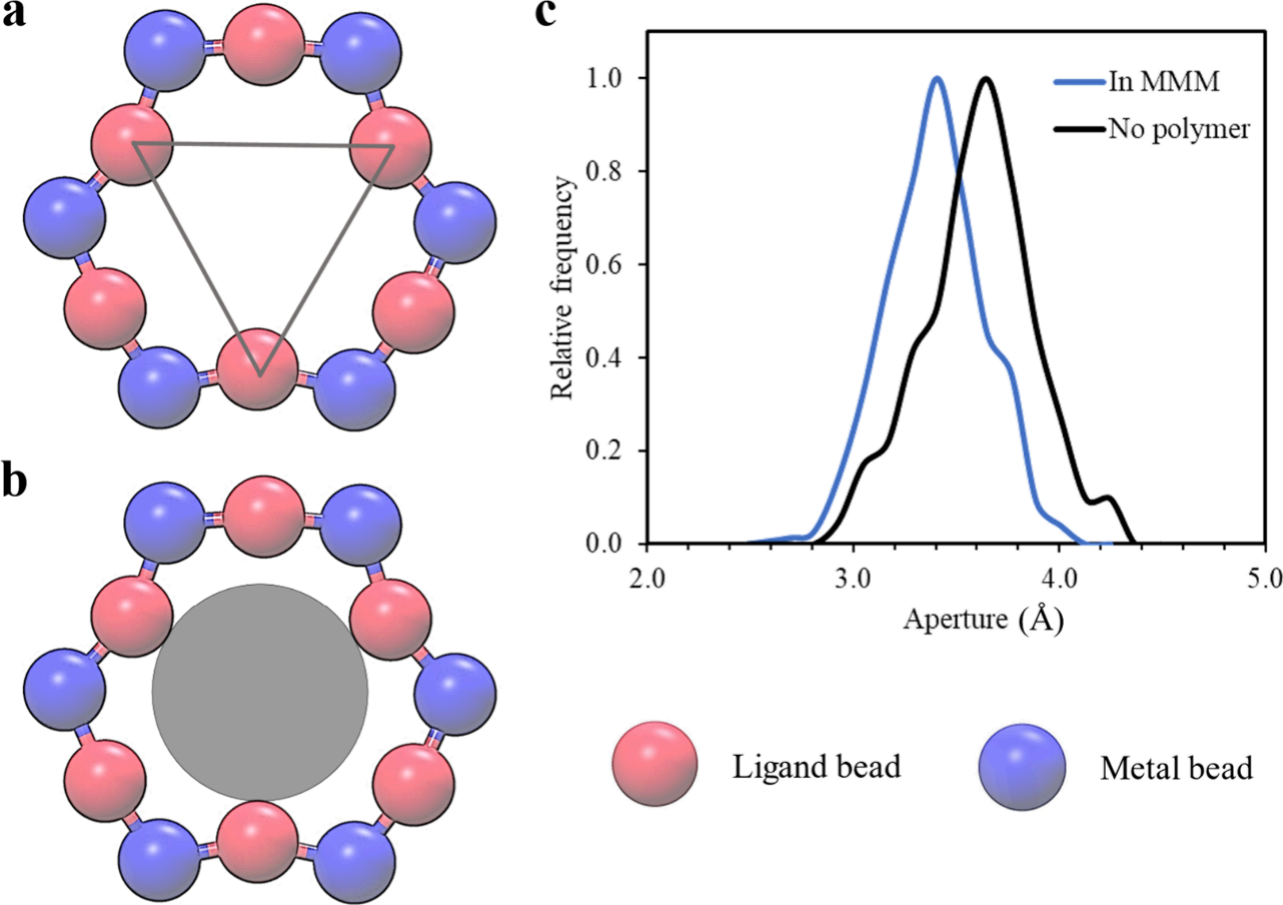
(a) Representative linker beads used to estimate geometrical diameter of the aperture. (b) Representation of the effective aperture after exclusion of the ligand bead radii,. (c) Aperture distribution of the smallest aperture in 2 nm particle MMM system.

Our investigation into the separation performance of C3 in ZIF-8 highlighted the significance of aperture size. We analyzed the distribution and frequency of apertures within nanoparticles, in both the presence and absence of a polymer matrix, from a qualitative standpoint. For the smallest nanoparticle size of 2 nm, we identified eight distinct aperture windows (Figure 8a,b). A comparative analysis of the smallest apertures among these revealed a notable reduction in both the mean aperture values and their standard deviation (Figure 8c). This suggests not only a decrease in aperture size in the presence of the polymer matrix but also a potential hindrance in angles and dihedrals, indicative of an induced pressurization effect on ZIF-8 apertures. The comparison between mean values, after the annealing process, for all apertures is systematically catalogued in Table S13.

Expanding our study to a larger nanoparticle, specifically a 3.6 nm particle, we observed a more complex structure with 64 apertures. To contextualize these findings, we differentiated apertures based on their proximity to the polymer matrix. Apertures near external cages exhibited mean values akin to those in ZIFs without polymer, albeit with a reduced standard deviation. Conversely, some external apertures demonstrated lower values, suggesting that connecting cages distant from polymer interactions perform similarly to pristine ZIFs (refer to Table S14). The variability of aperture size within the internal cage is lower than the variability observed among the external surface apertures. This analysis leads to an intriguing conclusion that smaller particles tend to enhance gas selectivity efficiency in these systems.

**Figure 9:**
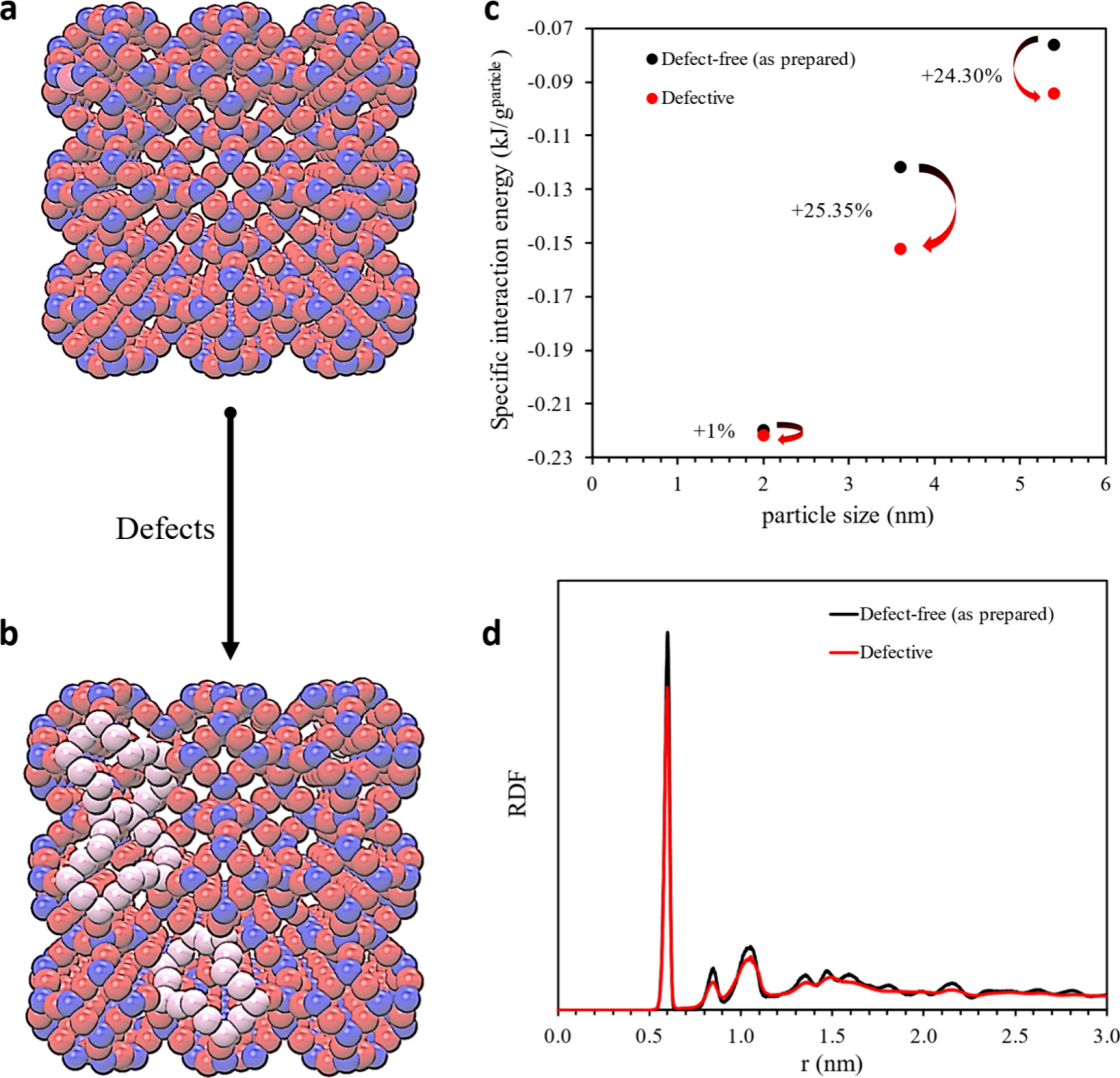
(a) 5.4 nm as prepared by the methodology. (b) Highlighted introduced defects. (c) Specific interaction energy as defects are introduced in the nanoparticle. (d) Radial distribution funciton of Zn–Zn in the nanoparticle of size 5.4 nm in MMM using defect-free (as prepared) and defected structure.

To elucidate the impact of amorphous-like nanoparticles on specific interaction energy, we introduced defects into nanoparticles of sizes 2, 3.6, and 5.4 nm. This was achieved by randomly removing about 7.5% and 6.5% of ligands from 3.6 and 5.4 nm structures (see Figure 9a,b). Subsequent geometrical optimization and analysis of the radial distribution function of metal beads revealed that these modified structures exhibit a more amorphous character compared to their defect-free (initial structure) counterparts (loss of long-range Zn order and peak intensity reduction in Figure 9d). Intriguingly, our calculations of specific interaction energy indicated an enhancement in interaction correlating with the increased amorphous nature of the particles within the MMM (Figure 9c). The enhancement of interfacial interactions can be attributed to the penetration of the polymer into the nanoporous architecture, alongside the polymer’s occupancy within created interfacial cavities. To demonstrate this, we investigated the spatial dynamics between the polymer and the MOF nanoparticle, comparing scenarios both with and without defects (Figure S13). A notable reduction in the separation distance between the COM of the polymer and that of the nanoparticle was observed in cases featuring defects. Furthermore, for nanoparticles measuring 3.6 and 5.4 nm, we report instances where the distances were less than half the size of the nanoparticle, suggesting a penetration of certain polymer chains.

Furthermore, the amorphous quality of ZIF-8, compounded by these structural defects, is anticipated to increase selectivity while diminishing permeability.^55^ The observed reduction in permeability in our experiments is likely attributable to either a decrease in aperture size or the emergence of defects leading to open metal sites and an amorphous structure. The latter has been previously demonstrated to significantly enhance gas selectivity.^56^

We have examined the structural properties of the polymer as influenced by the size of the nanoparticle at a constant filler composition. Intriguingly, the ratio of the mean squared radius of gyration to the mean squared end-to-end distance of the polymer did not exhibit a discernible correlation with the proximity to the MOF nanoparticles. Our research extended to a detailed analysis of the spatial distribution of polymer chains within the simulation box. This involved a comparative study of the aforementioned ratio in the bulk polymer and MMMs containing nanoparticles of various sizes. The findings, depicted through histograms in Figure S15, intriguingly reveal how this ratio varies with nanoparticle size, indicating a distinct influence of nanoparticle dimensions on polymer structure. Interestingly, our findings indicate a greater variation in structural properties with larger nanoparticle sizes. This observation could be attributed to the increased disruption of polymer chain organization and dynamics due to the presence of larger nanoparticles, which may introduce more significant physical barriers and alter the local environment of the polymer chains. Also, histograms shown in Figure S16 clearly show the mean distance from the polymer chain’s center of mass to the ZIF-8 nanoparticle surface is lower with smaller nanoparticle size, which is consistent with the exhibited interaction energy.

**Figure 10:**
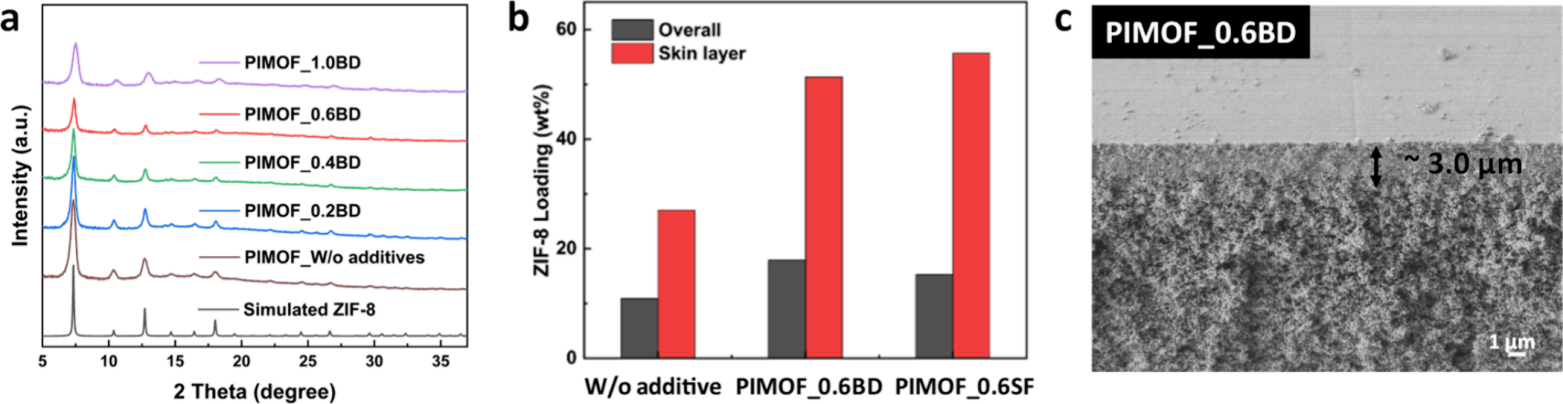
(a) XRD pattern of MMMs fabricated without additives and with 1,4-butanediol in the coagulation bath. (b) ZIF-8 loading percentages in the skin layer and all through the membranes. (c) Cross-section SEM image of PIMOF_0.6BD.

Our recent study revealed that 1,4-butanediol (hereafter BD) is an effective deprotonation agent to enhance the separation performances of polycrystalline ZIF-8 membranes when added into secondary growth solutions due to its basicity.^29^ Additionally, 1,4-butanediol promotes the preferentially oriented growth of ZIF-8 when the linker-to-metal ratio is lower than 16 in an aqueous growth solution. As such, we investigated 1,4-butanediol as an additive and compared its effects with those of sodium formate. Increasing the BD concentration in the coagulation bath substantiates the formation of ZIF-8, as confirmed by XRD analysis across all PIMOF_BD MMMs (Figure 10a). As with sodium formate, 1,4-butanediol promoted fast nucleation, resulting in a higher residue percentage as determined by TGA (Figures S1 and S2 and Table S1). The bulkier 1,4-butanediol relative to sodium formate poses challenges for its diffusion into the membrane matrix (i.e., diffusion-limited, rather than reaction-limited). As such, increasing concentrations of 1,4-butanediol facilitate its diffusion into the membrane, particularly into the porous layer with a larger diffusion length. Consequently, the overall loading percentage of ZIF-8 increases as 1,4-butanediol increases while it reaches a plateau in the case of sodium formate. The overall ZIF-8 loading in the PIMOF_0.6BD membrane was estimated to be approximately ca. 15.3 wt %, which is slightly lower than that of the PIMOF_0.6SF membrane (ca. 17.9 wt %) (Figure 10b).

**Figure 11:**
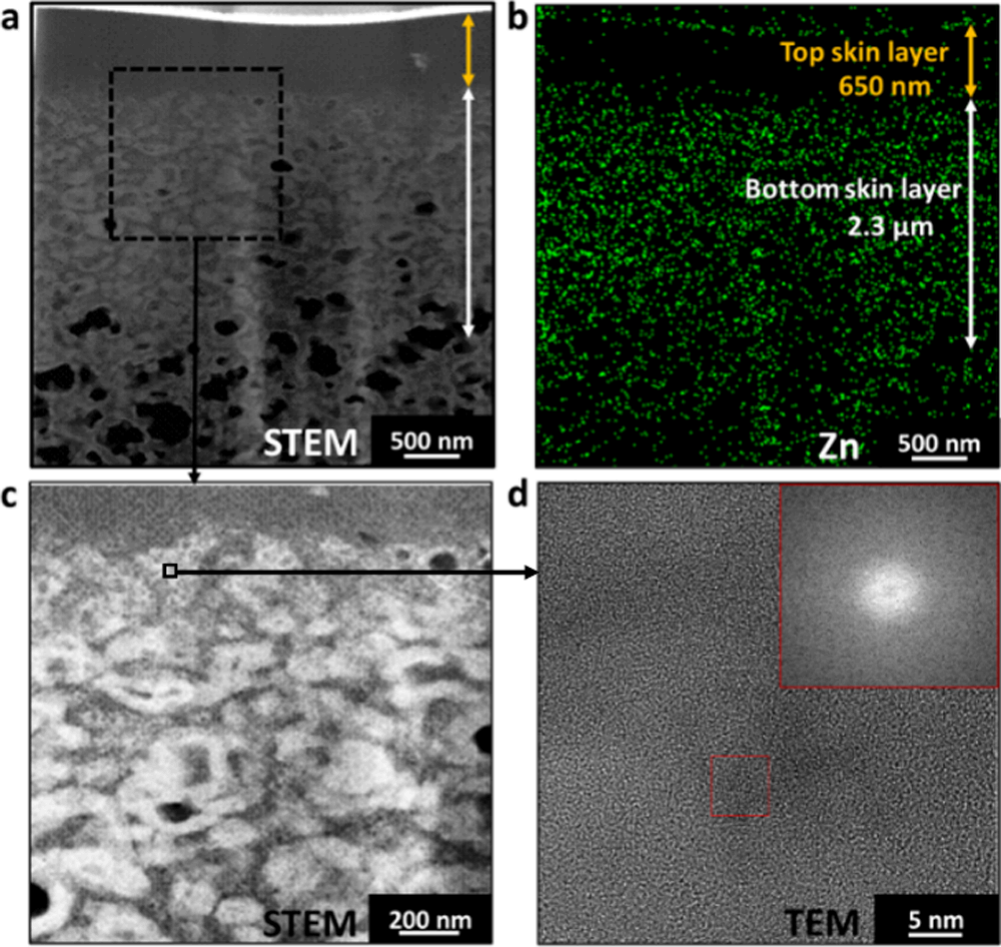
(a) STEM image of the cross-section of PIMOF_0.6BD. (b) Zinc EDS elemental mapping image of the same sample as shown in (a). (c) TEM image of the interface between the top skin layer and bottom skin layers. (d) HRTEM of the potential ZIF-8 particles and the corresponding FFT pattern (inset).

The microstructure of PIMOF_0.6BD was found to be similar to that of PIMOF_0.6SF. As shown in Figures 10c and 11a, it has an asymmetric structure with a ca. 3.0 μm thick apparent skin layer comprised of a ca. 650 nm thick top dense skin layer and ca. 2.3 μm thick bottom skin layer. Additionally, unlike in the case of PIMOF_0.6SF, TEM analysis reveals the presence of nanoparticles with a size of ca. 5 nm (indicated by the red square in Figure 11d). The corresponding fast Fourier transform (FFT) analysis suggests that the dark spot enclosed in the red square is a ZIF-8 particle with poor crystallinity. It is noted that, like PIMOF_0.6SF, a majority of particles appear unstable under TEM, indicating they are amorphous or poorly crystalline.

**Figure 12:**
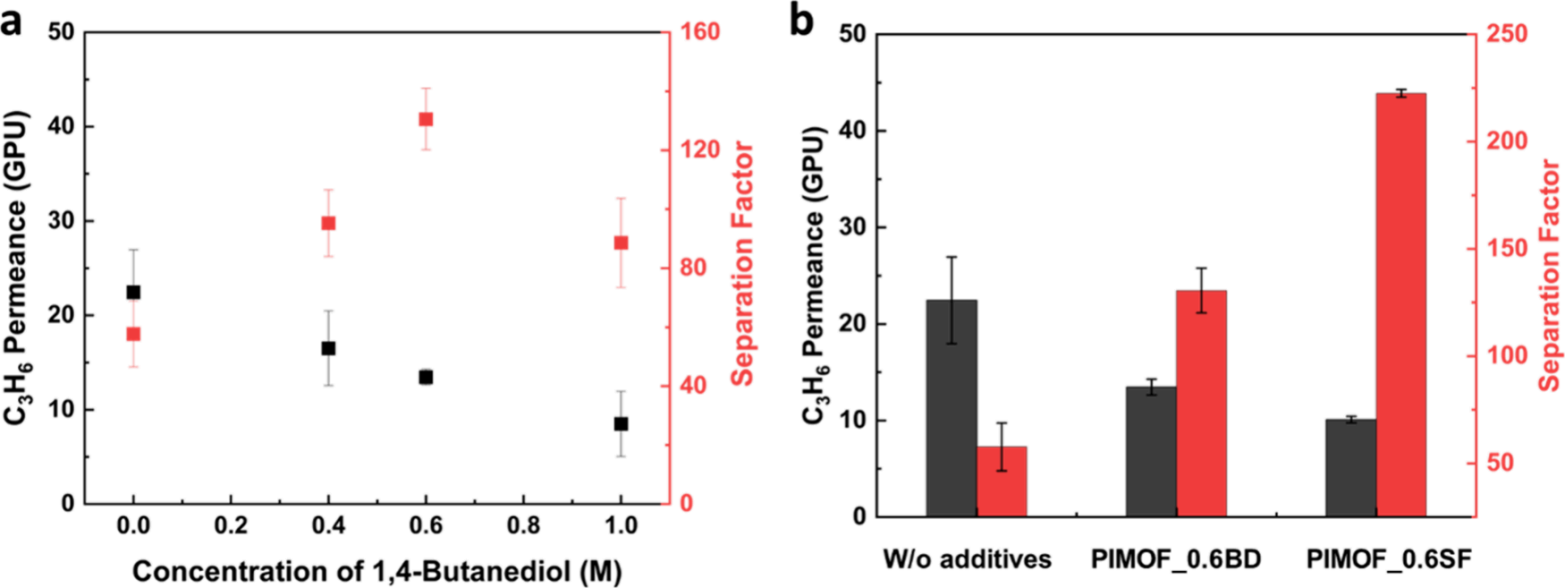
(a) The C3 separation performance of the PIMOF_MMMs with various concentrations of 1,4-butanediol in the coagulation bath. (b) The C3 separation performance of PIMOF_0.6BD with those of PIMOF_0.6SF and PIMOF_w/o additives.

In terms of gas separation performance, a similar trend was observed when 1,4-butanediol was used as an additive (Figure 12a and Table S2). With an increase in the butanediol concentration in the coagulation bath, the separation factor initially increased but eventually exhibited a downward trend, while the propylene permeance continued to decrease. Notably, the separation factor reached ca. 130.6 ± 10.4, which was approximately twice as high as that of membranes without additives (SF: ca. 57.7 ± 11.2) (Figure 12b). These results indicate the potential of 1,4-butanediol as an effective additive in enhancing the gas separation performance of PIMOF membranes. PIMOF_0.6BD exhibits a slightly higher propylene permeance but a much lower separation factor than PIMOF_0.6SF.

**Figure 13:**
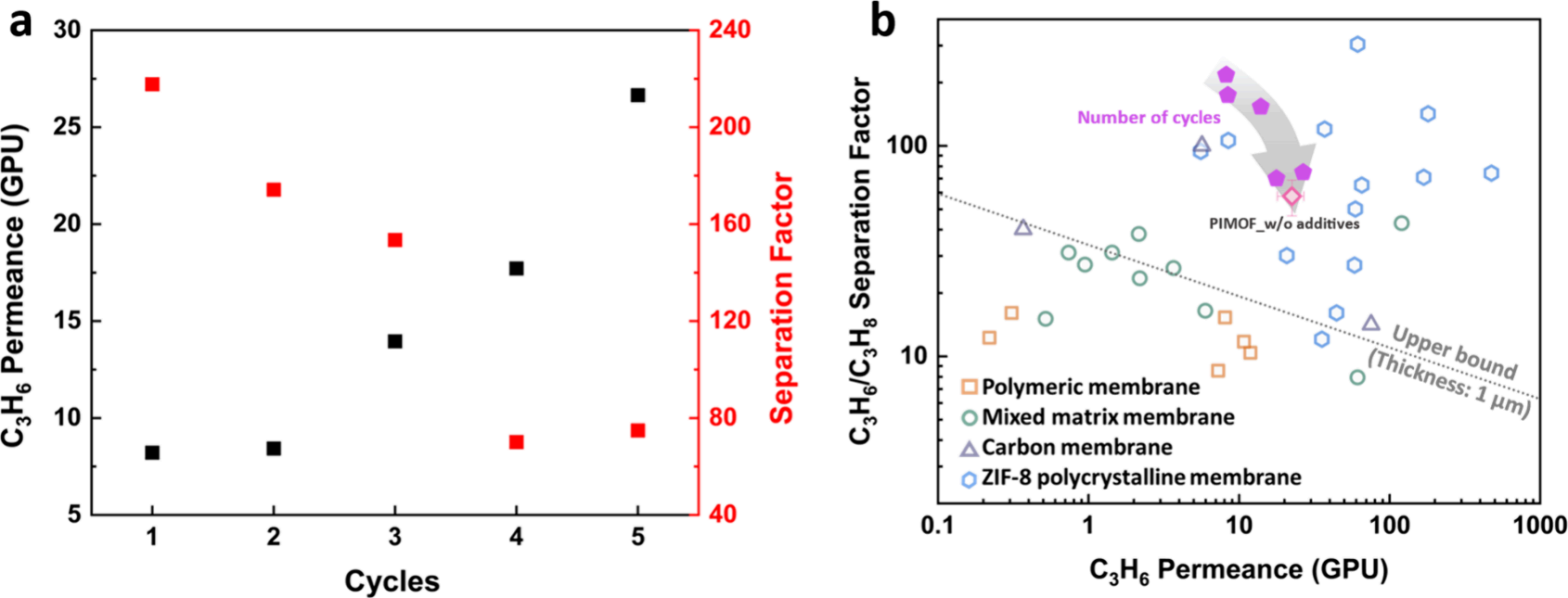
(a) The C3 separation performance of the PIMOF_MMMs with recycled coagulation bath. (b) The C3 separation performances of the PIMOF_MMMs with recycled coagulation bath in comparison with other membranes.

For the practical large-scale processing of PIMOF_MMMs, it is crucial to consider economic and environmental costs associated with chemical waste and reagent replacement. It was attempted to recycle the coagulation bath containing unreacted linkers, sodium formate, and solvent (water). The successful recycling of reagents would highlight the potential of the PIMOF process to transition into a continuous and scalable strategy for membrane fabrication. Figure 13 demonstrates the exceptional performance of the as-casted membrane even after undergoing five cycles of the phase inversion process without replenishing any reagents in the coagulation bath. The membrane retains its outstanding separation performance with a C_3_H_6_ permeance of ∼26.6 GPU and a separation factor of ∼74.9. This result showcases the robustness and stability of the PIMOF process and emphasizes its suitability for sustainable and cost-effective membrane production.

## Conclusions

Previously, we introduced an innovative and facile mixed-matrix membrane (MMM) fabrication technology known as the Phase-Inversion in Sync with MOF Formation (PIMOF). In this work, we have further advanced the PIMOF process by incorporating two distinct modulating additives, sodium formate and 1,4-butanediol, into the coagulation bath. The resulting membranes, PIMOF_0.6SF and PIMOF_0.6BD, exhibit remarkable C_3_H_6_/C_3_H_8_ separation factors of ca. 222.5 ± 1.8 and ca. 130.6 ± 10.4, respectively, alongside impressive C_3_H_6_ permeances of ca. 10.1 ± 0.4 GPU (flux: 3.8 × 10^–6^ m^3^/(m^2^ s)) and ca. 13.46 ± 0.82 GPU (flux: 5.1 × 10^–6^ m^3^/(m^2^ s)). The performances of the membranes, in particular, PIMOF_0.6SF, are comparable to, or even surpass, those of high-performance polycrystalline ZIF-8 membranes. The enhanced separation capabilities are attributed to the constrained linker-swing motion of exceedingly small amorphous or poorly crystalline ZIF-8-like filler particles formed within the confined environment of the polymer, facilitated by the presence of sodium formate as an additive. Furthermore, the as-casted membrane maintains its exceptional separation performance, with a C_3_H_6_ permeance of ca. 26.6 GPU and a separation factor of ca. 74.9, even after subjecting the coagulation bath to five cycles of the phase inversion process without replenishing reagents. This outcome underscores the enormous potential of the PIMOF technology for scalable membrane-based separation process.

The hypothesis, built on experimental data, was further validated through molecular simulations using CG models. The simulations showed that specific interactions between the polymer and ZIF-8 nanoparticles increase as the nanoparticle size decreases. Moreover, simulation indicates the hindrance of swing motion and reduction in variability of the aperture of ZIF-8 which leads to improved C3 selectivity. Through introducing defects in the nanoparticles, specific interactions increase as nanoparticles exhibit an additional degree of amorphicity. We believe that the combined effect of amorphous nature and smaller nanoparticle size is predominantly the reason for improved gas separation selectivity. In addition, the calculation of the polymer chain structure in MMM with several nanoparticle sizes shed light on the importance of studying the polymer structure, as some results indicated distinction in polymer structure at different nanoparticle sizes. In addition, our computational work provided predictive insights regarding the effect of filler loading in MMM for which no computational work has been presented previously in the literature. Overall, our study contributes to the growing body of knowledge on polymer–nanoparticle interactions, highlighting the size-dependent nature of these interactions and their impact on the structural properties of polymers. This research has potential implications for the design and optimization of nanocomposite materials.

## Supporting Information Available

Overall ZIF-8 loading percentage with various additive concentrations, TGA thermogram of samples under air flow, SEM images of cross-section of the PIMOF_MMMs, summary of C3 separation performances of PIMOF_MMMs, XRD pattern of the PIMOF_0.6SF MMMs with various acid treatment times, computational details, and results from molecular simulations (PDF)
