# Supplementary material for: Unexpectedly High Propylene/Propane Separation Performance of Asymmetric Mixed-Matrix Membranes through Additive-Assisted In Situ ZIF-8 Filler Formation: Experimental and Computational Studies
Source: ACS Appl Mater Interfaces. 2024 Mar 14;16(12):15273–85. doi: 10.1021/acsami.3c19491 (PMC10982995; doi:10.1021/acsami.3c19491)
Supplement: Supplementary file 1 — am3c19491_si_001.pdf [file am3c19491_si_001.pdf]

Supporting Information

Unexpectedly High Propylene/Propane Separation Performance of Asymmetric Mixed-Matrix Membranes through Additive-Assisted *In-Situ* ZIF-8 Filler Formation: Experimental and Computational Studies

Yinying Hua[a], Amro M. O. Mohamed[c], Gyeong Min Choi[d], Kie Yong Cho[d], Ioannis G. Economou[c], and Hae-Kwon Jeong*[,a,b]

[a] Artie McFerrin Department of Chemical Engineering and [b] Department of Materials Science and Engineering, Texas A&M University, 3122 TAMU, College Station, TX 77843-3122, United States

[c] Chemical Engineering Program, Texas A&M University at Qatar, PO Box 23874, Doha, Qatar

[d] Department of Industrial Chemistry, Pukyong National University, 45 Yongso-ro, Nam-gu, Busan 48513, Republic of Korea

** Corresponding author: H.-K. Jeong (e-mail address: hjeong7@tamu.edu, Phone: +1-979-862-4850, Fax: +1-979-845-6446)*

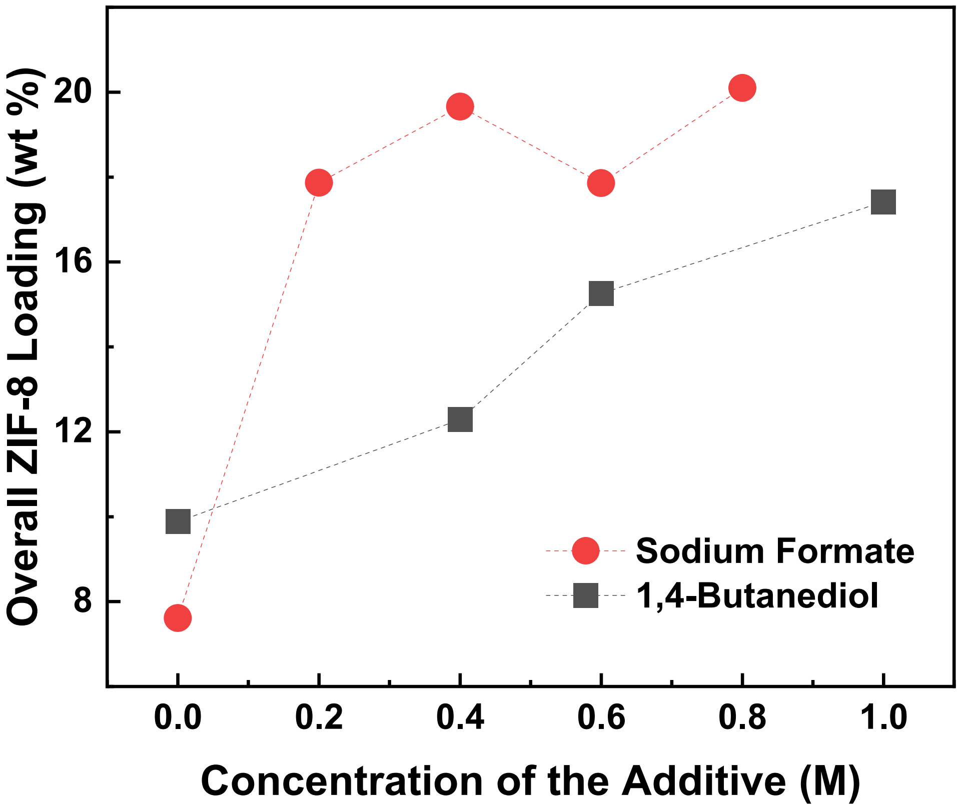

Figure S1. Overall ZIF-8 loading percentage with various additive concentrations

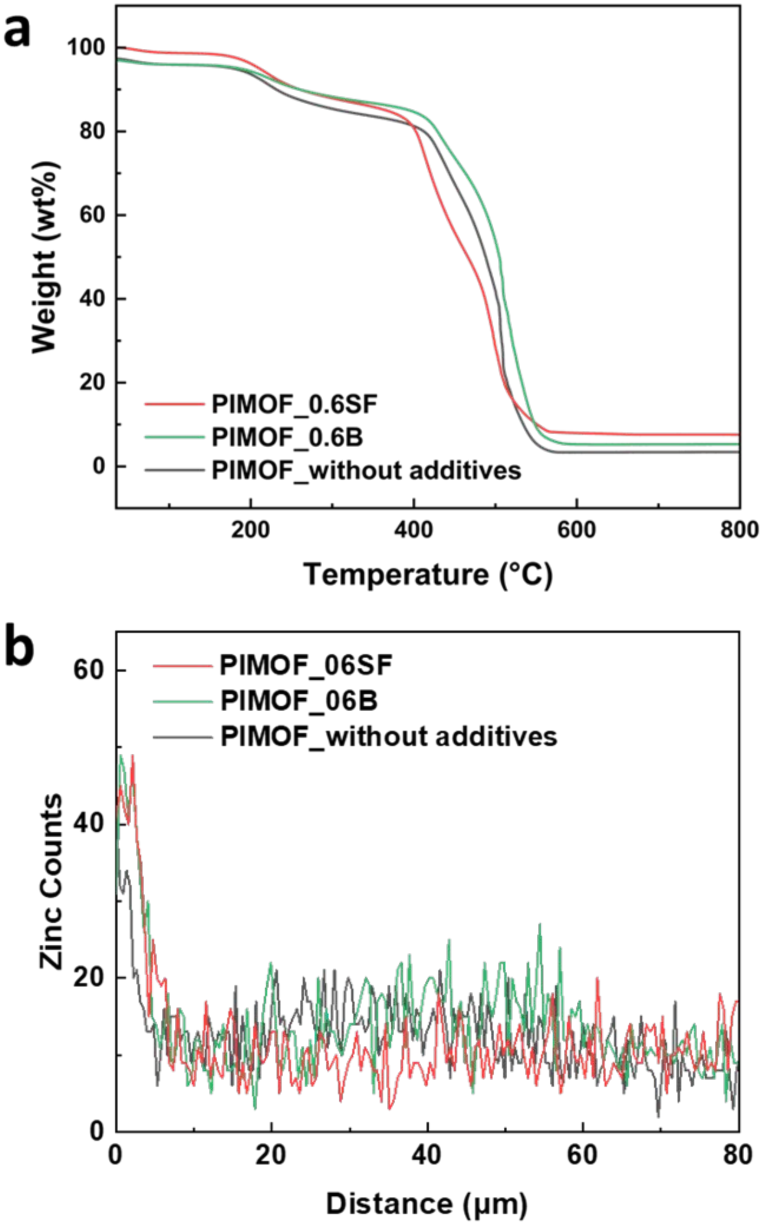

Figure S2. **a** TGA thermogram of samples under air flow; **b** Zinc counts of SEM-EDS line scanning of PIMOF_MMMs cross section

$$\mathbf{IF-8\ loading\ at\ a\ top\ 5\ um\ layer}\ (\mathbf{wt\%}) = \frac{\mathbf{Average\ Zinc\ counts}\ (\mathbf{top\ 5\ um\ layer})}{\mathbf{Average\ Zinc\ counts}\ (\mathbf{overall})} \times \mathbf{Total\ ZIF-8\ loading}\ (\mathbf{wt\%})$$

Table S1. Summary of overall ZIF-8 loading percentage and that in the apparent skin layer of the PIMOF_MMMS with/without additives

Additive		Overall ZIF-8 loading (wt%)	ZIF-8 loading at the top 3 um layer (wt%)
**Type**	**Concentration (M)**		
**None**		10.9	27.0
**Sodium formate**	0.6	17.9	51.3
**1,4-butanediol**		15.3	55.7

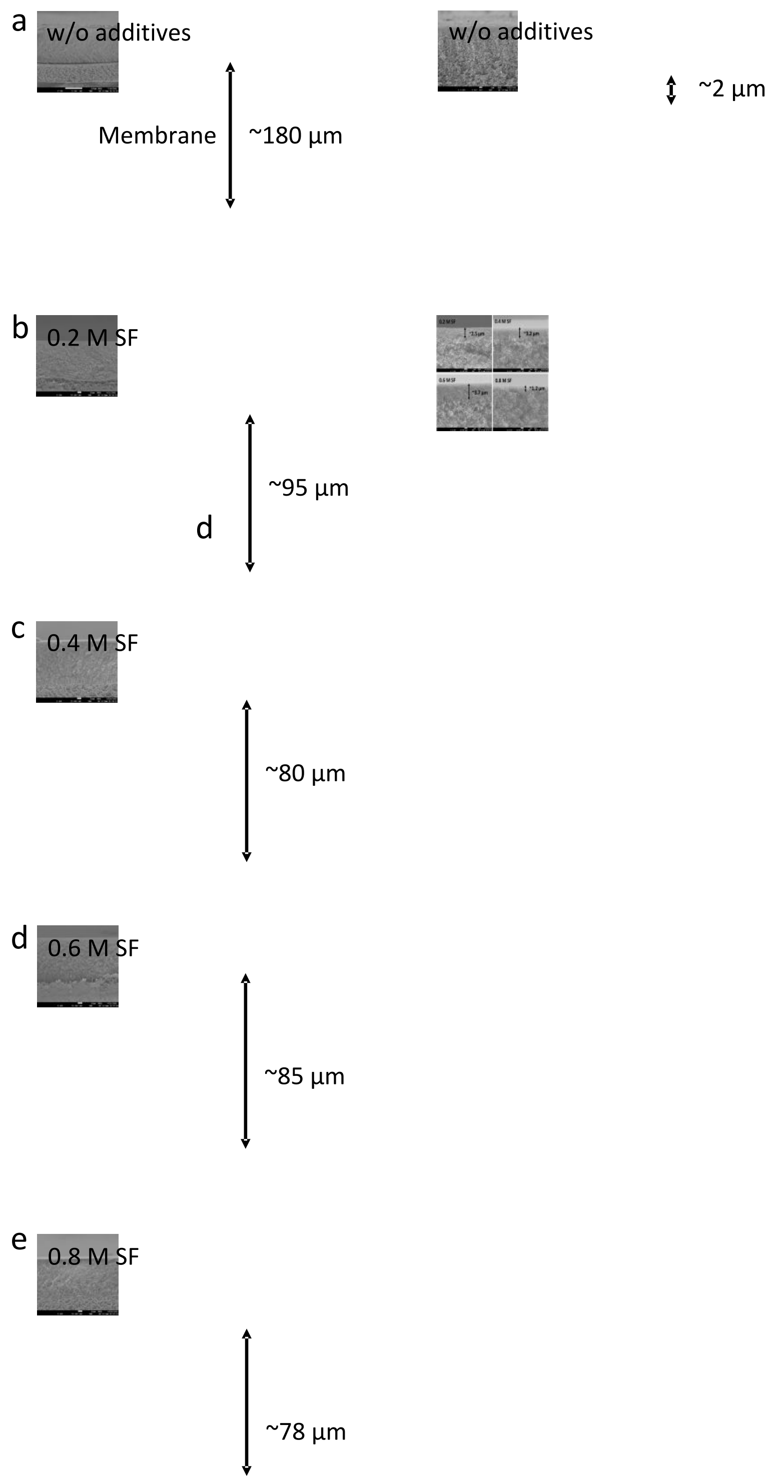

Figure S3. SEM images of cross-section of the PIMOF_MMMs with various concentrations of sodium formate

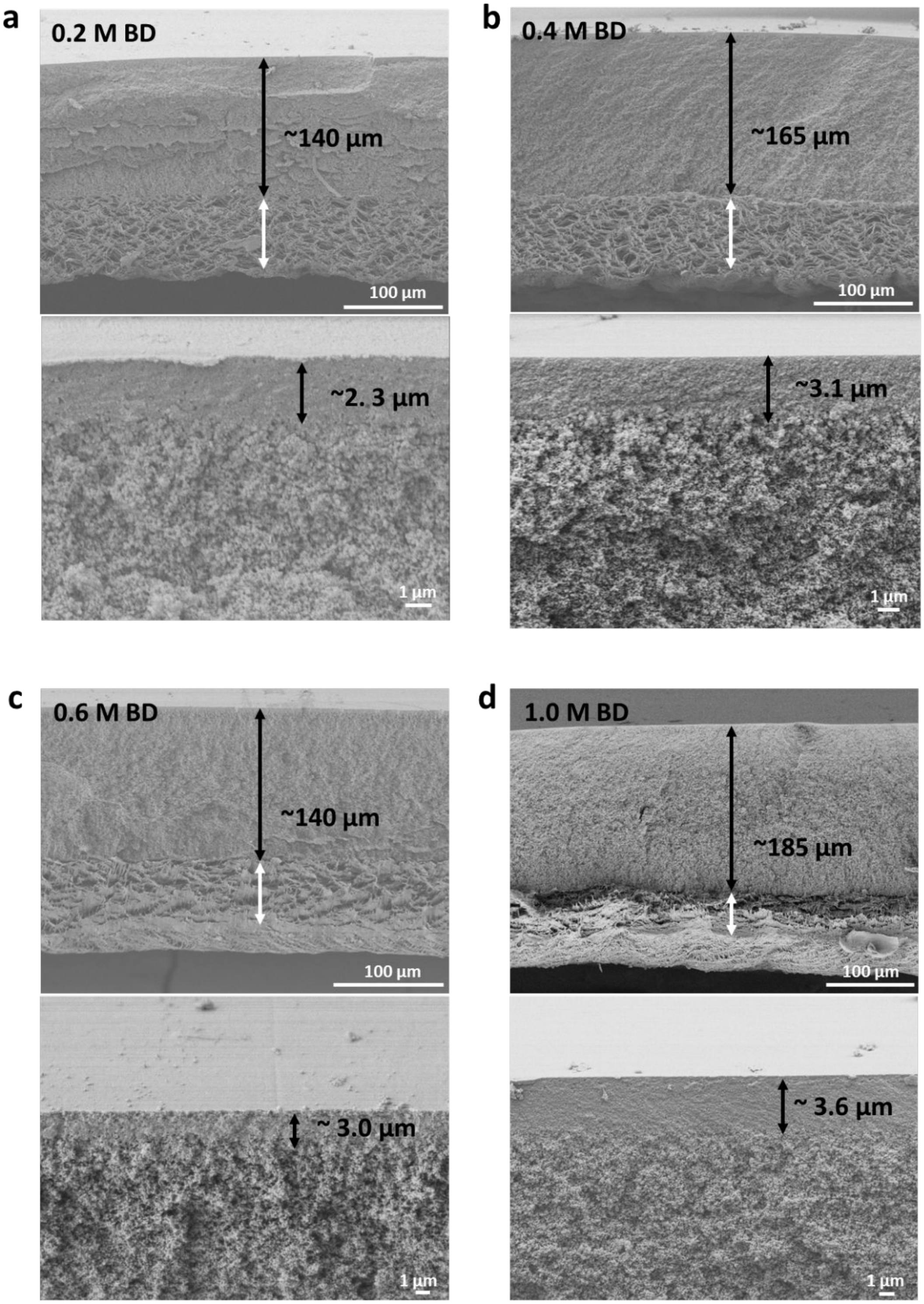

Figure S4. SEM images of cross-section of the PIMOF_MMMs with various concentrations of 1,4-butanediol

Table S2. Summary of C3 separation performances of PIMOF_MMMs measured using equimolar C3 gas mixture at ~ 1 atm and RT, all membranes were casted at RT and the HmIm concentration in the coagulation bath is 2 M.

Additive		Propylene permeance (GPU)	Propylene/propane separation factor
**Type**	**Concentration (M)**		
**None**		22.5 ± 4.5	57.7 ± 11.2
**Sodium formate**	0.2	11.5 ± 1.3	112.4 ± 27.5
	0.4	10.3 ± 0.4	177.5 ± 1.8
	0.6	10.1 ± 0.3	222.5 ± 1.8
	0.8	10.6	171.7
**1,4-butanediol**	0.4	16.5 ± 4.0	95.3 ± 11.3
	0.6	13.5 ± 0.8	130.6 ± 10.4
	1.0	8.5 ± 3.5	88.6 ± 15.1

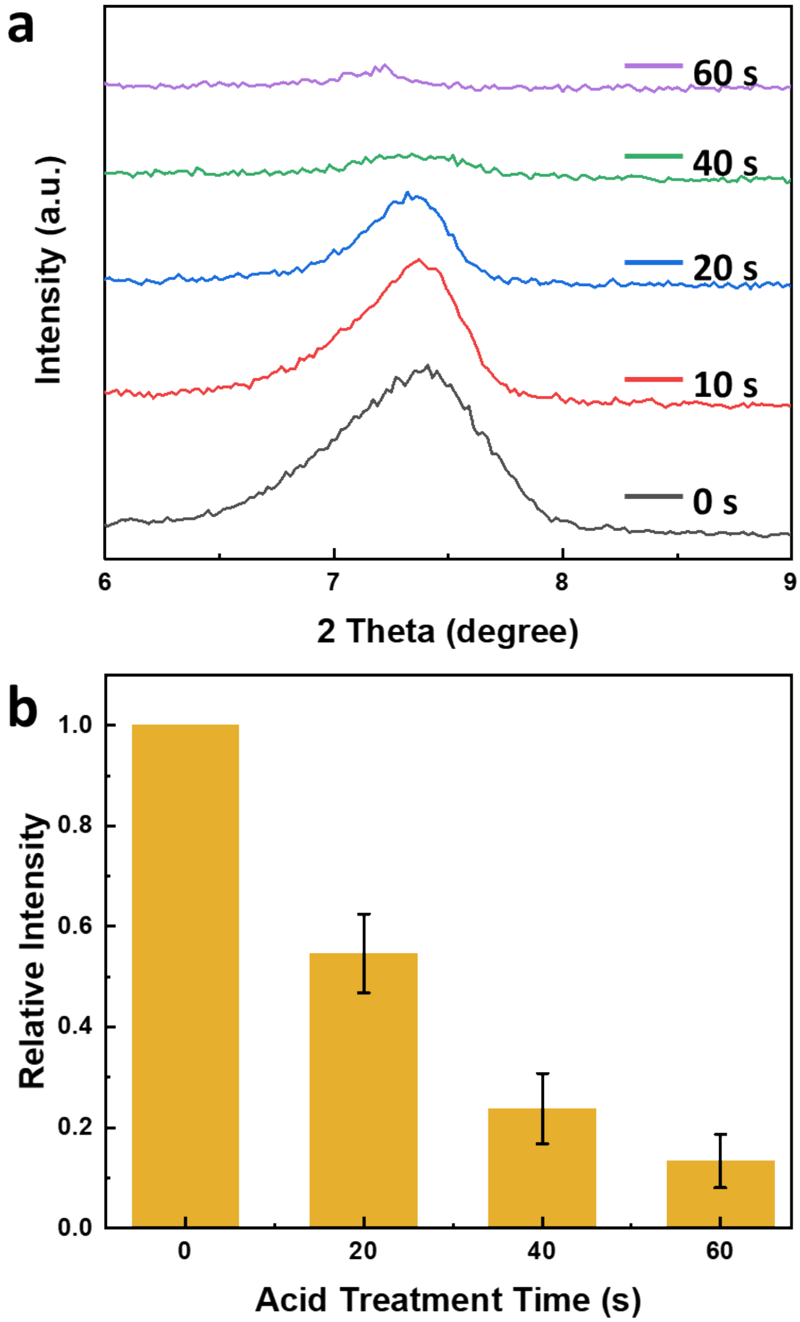

Figure S5. **a** XRD pattern of the PIMOF_0.6SF MMMs with various acid treatment time; **b** relative intensity of (110) peak of ZIF-8 pre and post acid treatment.

Computational details:

1. Force field for ZIF-8

1.1. Simulation details regarding force field optimization and testing for ZIF-8 Coarse-grained model

1.1.1. Non-bonded parameters

The non-bonded interactions are described by the Lennard-Jones (LJ) potential and the Coulombic potential for the charge-charge electrostatic interactions using the Ewald summation (Eq. S1).

$$U_{LJ}(r_1 \dots r_n) = \sum_{i,j} 4\varepsilon_{ij}\left[\left(\frac{\sigma_{ij}}{r_{ij}}\right)^{12} - \left(\frac{\sigma_{ij}}{r_{ij}}\right)^{6}\right] + \sum_{ij} \frac{q_i q_j}{4\pi\varepsilon_0 r_{ij}} \quad \text{(S1)}$$

Two beads were assigned in the CG for ZIF-8. One to represent the atom (Zn) called bead M and one to represent the linker (2-methylimidazole) called bead L. The LJ parameters for bead M were transferred from atomistic values since this is a 1:1 mapping using the CHARMM22 parameter set modified by Stote and Karplus for divalent zinc [1]. The CHARMM22 parameters for Zn are expected to offer a better representation of Zn sites when dealing with defects and open metal sites in ZIF-8 as other generic force fields (FF, such as UFF and DREIDING) showed inaccurate adsorption values in open metal MOFs because of failure to characterize specific interactions between adsorbates and metal site of the MOF [2]. The selected zinc parameters permit different coordination geometries and ligand exchange for the zinc ion and were tested in molecular dynamics simulations of two zinc metalloenzymes, namely carboxypeptidase A and carbonic anhydrase. To the best of our knowledge, this is the first time that CHARMM22 parameters are used for zinc atoms. We believe that this would identify correctly interactions with solvents and

other entities at amorphized and defected ZIF-8; while not affecting greatly the properties of pristine ZIF-8.

The energy parameter for bead L was adjusted by obtaining agreement with available CO_2 Henry's coefficient using the Widom insertion method implemented in Monte Carlo simulation in RASPA2 code [3]. No further adjustments to the LJ parameters were made. The charges for the two beads were assigned using atomistic charges based on the work of Economou and coworkers [4]. The charge assigned for Zn is used to represent the M bead whereas the L was assigned the charge to make the framework neutral (alternatively, it can be done by adding up the charges of the atoms building the bead). We excluded interactions between interaction sites up to 1 bond away, known as 1-2 interactions. In summary, non-bonded interaction parameters are shown in Table S3.

Table S3. Non-bonded interaction parameters for the CG model of ZIF-8

	ZIF-8 constitute	Bead type	Mass (Da)	ε/k_B [K]	σ [Å]	q [e]
1	Metal (Zn)	M	65.08	149	1.96	0.70
2	Linker (2-methylimidazole)	L	81.00	180	5.32	-0.35

1.1.2. Bonded parameters

We used FF functional forms as implemented in atomistic FFs for ease of implementation in all available software and codes. Bond stretching and bond angle bending are described by harmonic potentials as shown in Eqs S2 and S3. The torsional potential in the framework is given by Eq. S4. Simulations to optimize the CG FF were made possible by using GROMCAS software [5] at the

NpT ensemble using MD simulations with a time step of 2 fs. The thermostat and barostat used temperature coupling using velocity rescaling and Berendsen.

$$U_b(r_{ij}) = \frac{1}{2}k_{ij}^{b}(r_{ij} - b_{ij})^2 \tag{S2}$$

$$U_a(\theta_{ijk}) = \frac{1}{2}k_{ijk}^{\theta}(\theta_{ij} - \theta_{ij}^{0})^2 \tag{S3}$$

$$U_t(\phi_{ijkl}) = k_{\phi}(1 + \cos(m\phi - \phi_0))^2 \tag{S4}$$

Table S4. Bonded interaction parameters for the ZIF-8 FF1 CG model

Bond Type	$\boldsymbol{k_{ij}^{b}}$ (kJ mol^{-1} nm^{-2})	$\boldsymbol{b_{ij}}$ (Å)
M – L	60000	2.95
Angle Type	k_{ijk}^{θ} (kJ mol^{-1} rad-^{2})	θ_{ij}^{0}
M – L – M	120	166.6
L – M – L	92	117.8
Dihedral Type	k_{ϕ}(kJ mol^{-1})	ϕ_0, m
L – M – L – M	0.088	0, 3

1.2. Results associated with CG modeling of ZIF-8

Table S5. Heat of adsorption of various adsorbates in ZIF-8 using CG FF

**Adsorbate**	**Heat of adsorption at 298 K and 1 bar**
**CO_2**	-15.33 ± 0.05

**CH_4**	-10.69 ± 0.03
**N_2**	-9.05 ± 0.02
**H_2**	-5.20 ± 0.01
**NH_3**	-20.0 ± 0.2
**C_2H_4**	-14.06 ± 0.03
**C_2H_6**	-16.1 ± 0.1
**C_3H_6**	-23.2 ± 0.2
**C_3H_8**	-24.9 ± 0.2

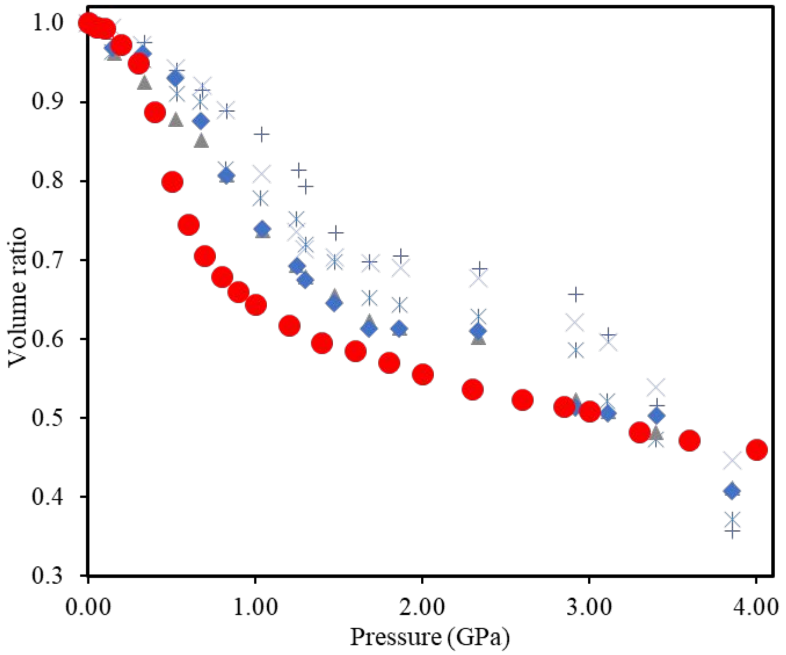

Figure S6. Volume ratio change as a function of pressure (up to 4 GPa) and comparison with experimental measurements by Zhi et al. [6]. Comparison with experimental work by Chapman et al. [7] shows similar amorphization starting at approximately 0.34 GPa (not shown here).

2. Force field for 6FDA-DAM

2.1. Simulation details regarding force field optimization and testing for 6FDA-DAM Coarse-grained model

Initial configurations were prepared following a simulation procedure that employs an annealing process. The annealing process was adopted to ensure a local energy minimization of the glassy polymer. The process starts with a few energy minimizations followed by NVT thermalization at 300 K for 1 ns. After that, an NVE heating (from 300 K to 800 K) is accomplished at a rate of 250 K/ns. The process of cooling used is a slow process that includes intermittent NPT simulations. Cooling is done at 25 K/ns, followed by NPT equilibration at the reached temperature to estimate the density and equilibration of the structure. This is followed until the temperature reaches back to 300 K. The FF for the adsorbate was based on TraPPE.

2.2. Force field parameters to model 6FDA-DAM

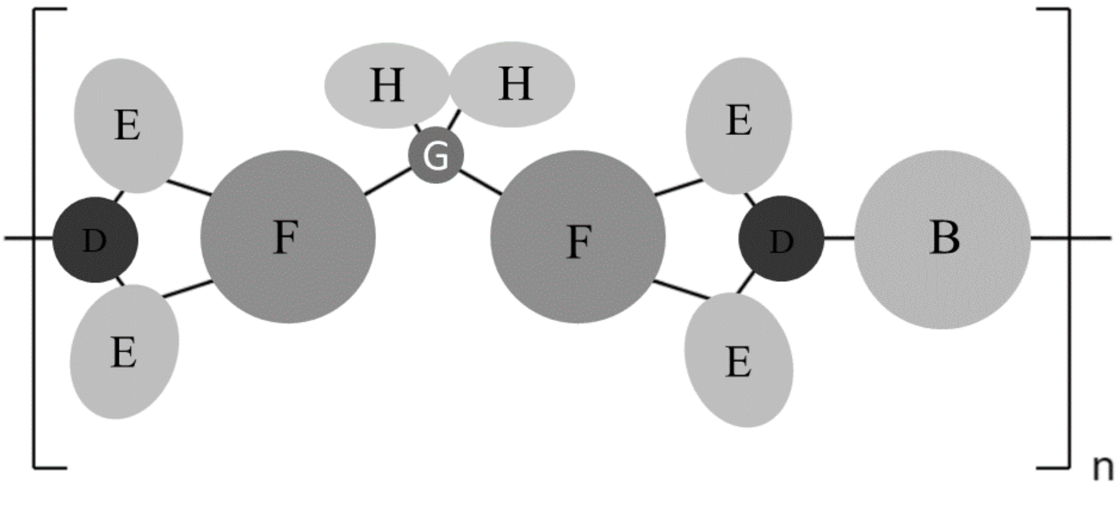

Figure S7. Bead identification for the polyimide, bead B represents the diamine co-monomer.

Table S6. Masses of beads used in the simulations.

Bead	Mass (*g mol⁻¹*)
**D**	15
**E**	28
**F**	75
**G**	12
**H**	69
**B**	132.2

Force field functional forms:

1- Bond stretching:

$$U_b(r_{ij}) = \frac{1}{2}k_{ij}^{b}(r_{ij} - b_{ij})^2 \qquad \text{(S5)}$$

2- Bond angle bending:

$$U_a(\theta_{ijk}) = \frac{1}{2}k_{ijk}^{\theta}(\theta_{ij} - \theta_{ij}^{0})^2 \qquad (S6)$$

3- Dihedral angle distortion:

$$U_{rb}(\phi_{ijkl}) = \sum_{n=0}^{5} C_n(\cos(\psi))^n ; \psi = \phi - 180 \; (S7)$$

4- Non-bonded interactions:

$$U_{LJ}(r_1 \dots r_n) = \sum_{i,j} 4\varepsilon_{ij}\left[\left(\frac{\sigma_{ij}}{r_{ij}}\right)^{12} - \left(\frac{\sigma_{ij}}{r_{ij}}\right)^{6}\right] \qquad \text{(S8)}$$

$$\sigma_{ij} = \left(\frac{\sigma_{ii} + \sigma_{jj}}{2}\right) - s_{ij} \ \ ; s_{ij} = 0.01 \qquad \text{(S9)}$$

s_{ij} is the softness parameter. The parameter assumes that bigger beads should be softer than smaller ones. ε_{ij} parameters based on the fully revised interaction matrix by the Martini 3 model and scaled for very large beads.

Table S7. Bonded interactions for the CG polyimides (see eq S5)

**Type**	k^{b}_{ij} ***(kJ mol^{-1} nm^{-2})***	b_{ij} (Å)
**D – E**	325694	1.85
**E – F**	345124	3.32
**F – G**	744542	2.92
**G – H**	403687	2.02
**B – D**	744542	2.836

Table S8. Bonded interactions for the CG polyimides (see eq S6)

**Type**	k^{θ}_{ijk} ***(kJ mol^{-1} rad^{-2})***	θ^{0}_{ij} **(º)**
**D – E – F**	2954	74
**E – F – G**	750	116
**F – G – H**	2547.36	121
**F – G – F**	1770.64	90

**E – F – E**	5089	63
**E – D – E**	1263.3	146
**E – D – B**	633.13	106
**D – B – D**	1195.87	178.04

Table S9. Bonded interactions for the CG polyimides (see eq. S7)

**Type**	**C_1 ($kJ\ mol^{-1}$)**	**C_2 ($kJ\ mol^{-1}$)**	**C_3 ($kJ\ mol^{-1}$)**	**C_4 ($kJ\ mol^{-1}$)**	**C_5 ($kJ\ mol^{-1}$)**	**C_6 ($kJ\ mol^{-1}$)**
**F – E – D – B**	4.01	-6.98	-3.33	9.58	2.14	-5.44
**E – D – B – D**	0.92	-0.13	-2.92	0.17	2.40	0.02
**G – F – E – D**	0.86	-0.16	4.33	2.20	-3.82	-3.06
**F – G – F – E**	0.41	1.26	2.88	-5.04	-2.41	4.67
**H – G – F – E**	0.58	-1.15	-0.25	2.20	0.18	-1.09

Table S10. Non-bonded interactions for the CG polyimides (6FDA-DAM)

**Type 1**	**Type 2**	**ε_{ij} ($kJ\ mol^{-1}$)**	**σ_{ij} (Å)**
**B**	D	2.790	5.048
**B**	E	2.790	5.048
**B**	F	6.131	5.988
**B**	G	2.109	4.818
**B**	H	4.529	5.338

**Type 1**	**Type 2**	ε_{ij} ***(kJ mol^{-1})***	σ_{ij} ***(Å)***
**B**	B	7.717	6.775
**D**	E	3.018	3.400
**D**	F	2.365	4.300
**D**	G	0.310	3.355
**D**	H	2.102	3.650
**D**	D	2.015	3.400
**E**	F	2.365	4.300
**E**	G	0.310	3.355
**E**	H	1.755	3.650
**E**	E	2.015	3.400
**F**	G	1.610	4.130
**F**	H	3.377	4.650
**F**	F	4.368	5.400
**G**	H	1.224	3.480
**G**	G	1.066	3.060
**H**	H	2.223	4.100

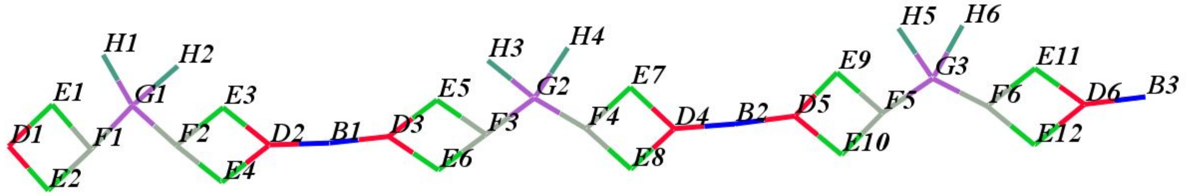

Figure S8. Atom types used to model 6FDA-DAM charges

Table S11. Charges used for the CG polyimides (trimer of 6FDA-DAM)

**Type**	**Charge (e)**	**Type**	**Charge (e)**	**Type**	**Charge (e)**
**D1**	-0.05007	D3	-0.14362	D5	-0.14362
**E1**	-0.03463	E5	-0.02687	E9	-0.02687
**E2**	-0.03464	E6	-0.02688	E10	-0.02688
**F1**	0.10996	F3	0.11117	F5	0.11117
**G1**	0.20952	G2	0.20952	G2	0.20952
**H1**	-0.09538	H3	-0.09538	H5	-0.09538
**H2**	-0.09538	H4	-0.09538	H6	-0.09538
**F2**	0.11117	F4	0.11117	F6	0.11117
**E3**	-0.02687	E7	-0.02687	E11	-0.02687
**E4**	-0.02688	E8	-0.02688	E12	-0.02688
**D2**	-0.14362	D4	-0.14362	D6	-0.14367
**B1**	0.15366	B2	0.15366	B3	0.07689

2.3. Results from the 6FDA-DAM CG model

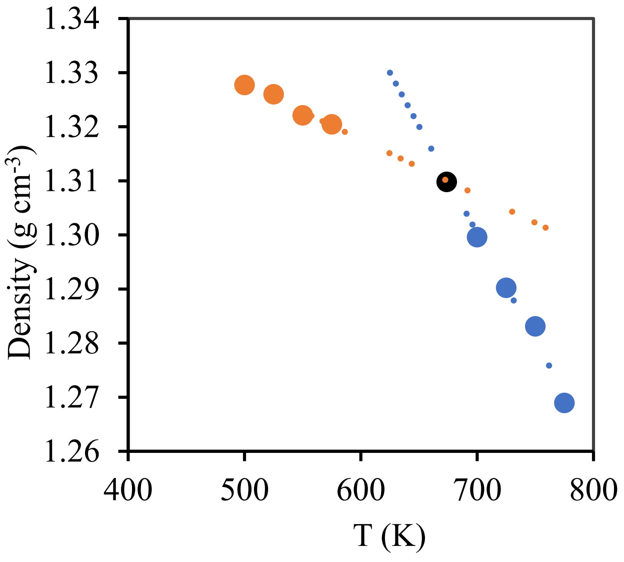

Figure S9. Density of 6FDA-DAM PI calculated from MD simulations at 1 bar. The black point represents the estimated T_g.

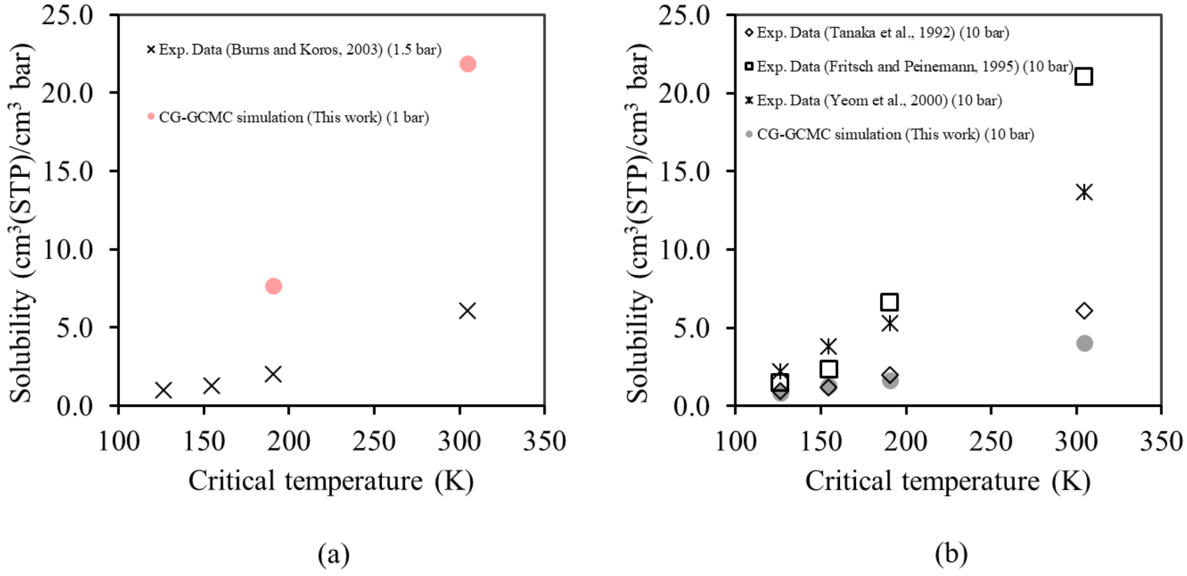

Figure S10. Experimental data and molecular simulation values for CO_2, CH_4, N_2 and O_2 solubility in 6FDA-DAM at 308 K and **a** low pressure and **b** high pressure as a function of the critical temperature of the gas.

3. Complementary details of the computational work on MMM using CG models

3.1. Structural properties

Structural properties of ZIF-8 such as pore diameters and pore size distribution (PSD) used to reflect on pore dimensionality and aperture size were estimated using zeo++ code. XRD of the atomistic and CG structures was found using Mercury software by CCDC [8]. The evaluation of the geometrical value of apertures was determined using r_{min}= 6.4 Å.

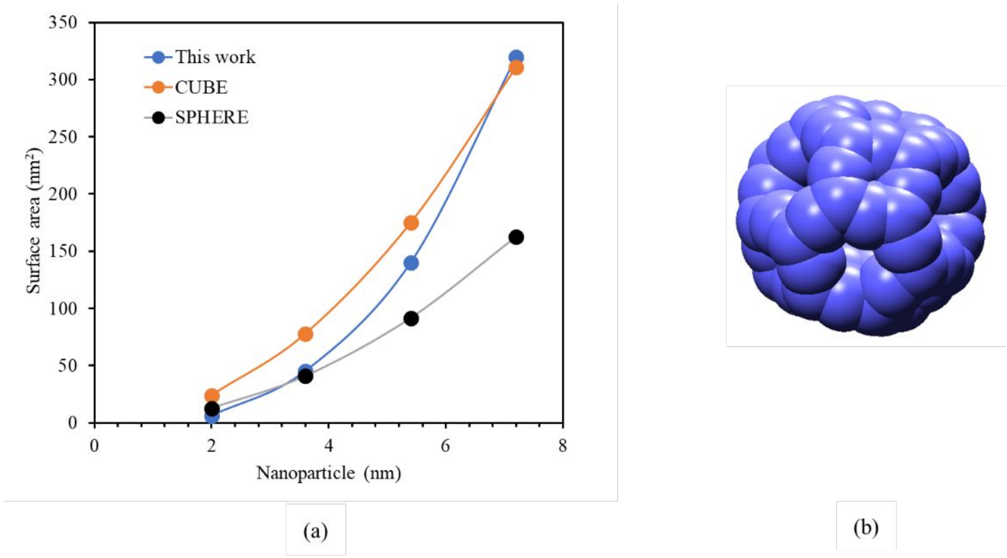

Figure S11. a Comparison between the estimated surface area of nanoparticle and simple approximation using sphere and cubic representations, **b** structure of the 2 nm particle

Additionally, we conducted a study on 2 nm particles (10 wt %), individually assessing each polymer chain to estimate adsorption (or adhesion) energy in the context of MMMs. The objective was to plot an interaction energy profile and identify the peak adhesion value correlating with the given nanoparticle structure. Figure S11 presents a subset of polymer chains and their interaction

with the nanoparticle interactions as a function of the distance (δ), defined as the separation between the respective center of masses (MOF-polymer).

Table S12. Nanoparticle self-diffusion order of magnitude as a function of size. The diffusivity of nanoparticles is indicative of mobility in the MMM composite.

Size (nm)	Self-diffusivity ($cm^2\ s^{-1}$)
**2.0**	$1.1 \times 10^{-12} \pm 3.2 \times 10^{-12}$
**3.6**	$9.7 \times 10^{-10} \pm 4 \times 10^{-11}$
**5.2**	$1.5 \times 10^{-7} \pm 6 \times 10^{-8}$
**7.2**	$2.6 \times 10^{-7} \pm 1.4 \times 10^{-7}$

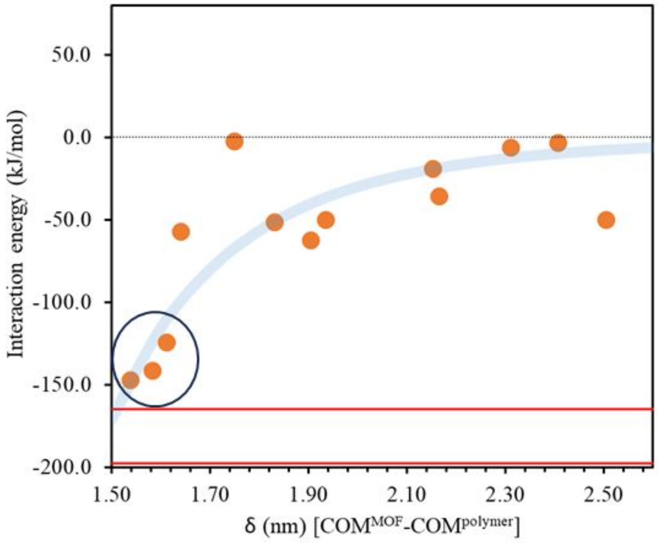

Figure S12. The interaction energy of individual polymer chains with the nanoparticle of size 2 nm inserted in 10 wt. % MMM. The light blue is the best-fitted line using a power law. The two

red horizontal lines are adhesion energy estimated from atomistic molecular simulation of MOF-based MMM systems for comparison basis [9].

We compared these energies with existing literature, such as Ghalei et al.'s computational exploration of enhanced adhesion in functionalized MOF-based MMM (UiO-66-NH_2 and PIM-1 system) [9]. The comparison revealed similarities in adhesion energy magnitudes. Our findings, particularly for polymer chains close to the nanoparticle surface (Figure S12, circle, ~-150 kJ/mol), compare well with the adhesion energy values reported in previous studies (-169 to -198 kJ/mol). Additional work is necessary to improve the accuracy of the CG force fields.

Table S13. Aperture details on the 2 nm particle size in isolation and 10 wt% MMM after annealing process. Details include the mean and standard deviation of the aperture and the range of the aperture.

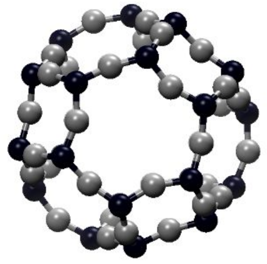

Aperture	In absence of the polymer			In the presence of the polymer		
	**Mean (Å)**	**Standard deviation (Å)**	**Range (Å)**	**Mean (Å)**	**Standard deviation (Å)**	**Range (Å)**
**1**	3.58	0.29	2.70-4.39	3.10	0.25	2.33-4.02

Aperture	In absence of the polymer			In the presence of the polymer		
	**Mean (Å)**	**Standard deviation (Å)**	**Range (Å)**	**Mean (Å)**	**Standard deviation (Å)**	**Range (Å)**
**2**	3.58	0.27	2.80-4.49	3.45	0.22	2.77-4.12
**3**	3.57	0.29	2.78-4.41	3.70	0.22	2.99-4.39
**4**	4.32	0.49	3.06-5.63	4.18	0.33	3.05-5.19
**5**	3.55	0.27	2.68-4.20	3.73	0.22	2.99-4.48
**6**	3.57	0.26	2.75-4.48	3.36	0.24	2.70-4.10
**7**	3.60	0.28	2.80-4.39	3.51	0.25	2.54-4.10
**8**	3.57	0.27	2.80-4.28	3.53	0.21	2.82-4.11

Table S14. Aperture details on the 3.6 nm particle size in MMM of 10wt% after the annealing process. Details include the mean and standard deviation of the aperture and the range of the aperture. The comparison is made by comparing samples of external apertures (near polymer matrix) and internal aperture (far from the polymer matrix, center of nanoparticle with Zn:L ratio of 4-1.

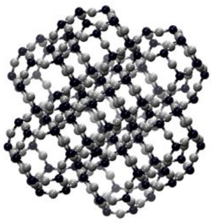

		In the presence of the polymer		
**Type**	**Aperture**	**Mean (Å)**	**Standard deviation (Å)**	**Range (Å)**
External Surface	**1**	3.22	0.28	2.45-3.90
	**2**	3.51	0.24	2.79-4.23
	**3**	3.39	0.25	2.52-4.14
	**4**	**3.12**	**0.26**	**2.24-3.89**
	**5**	3.69	0.23	3.00-4.30
Internal Core	**1**	3.60	0.25	2.84-4.34
	**2**	3.68	0.22	3.07-4.38
	**3**	3.65	0.22	3.07-4.30
	**4**	**3.53**	**0.23**	**2.82-4.33**

	**5**	3.64	0.21	2.85-4.37

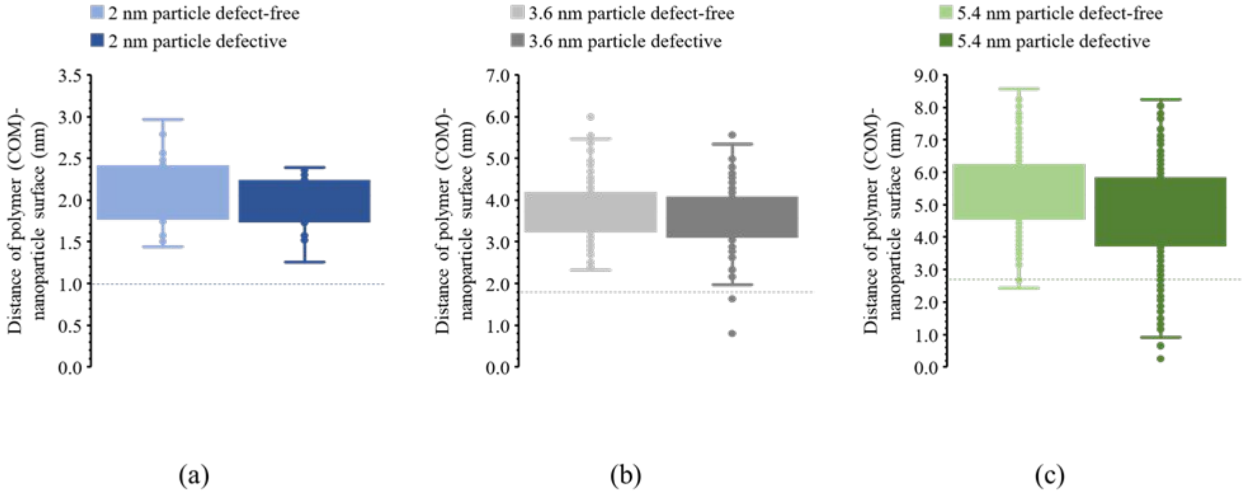

Figure S13. Distribution of distance of polymer COM to particle surfaces as a function of particle size (nm) in 10wt% filler composition.

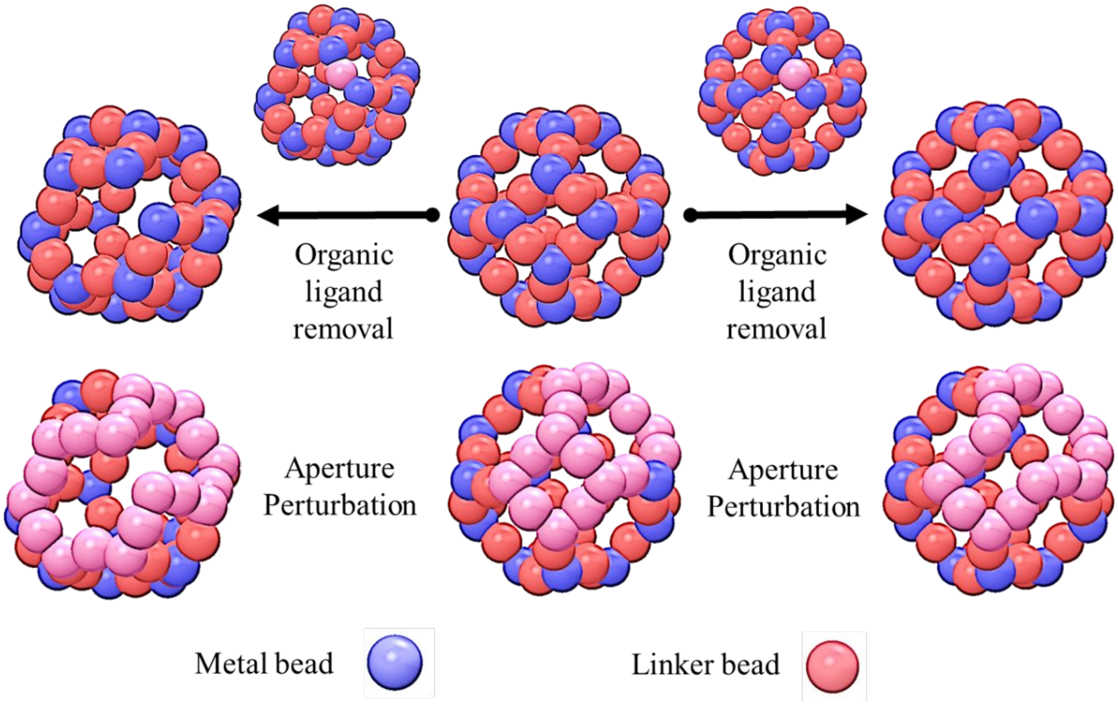

Figure S14. Example of aperture dimensions change by removing one organic ligand.

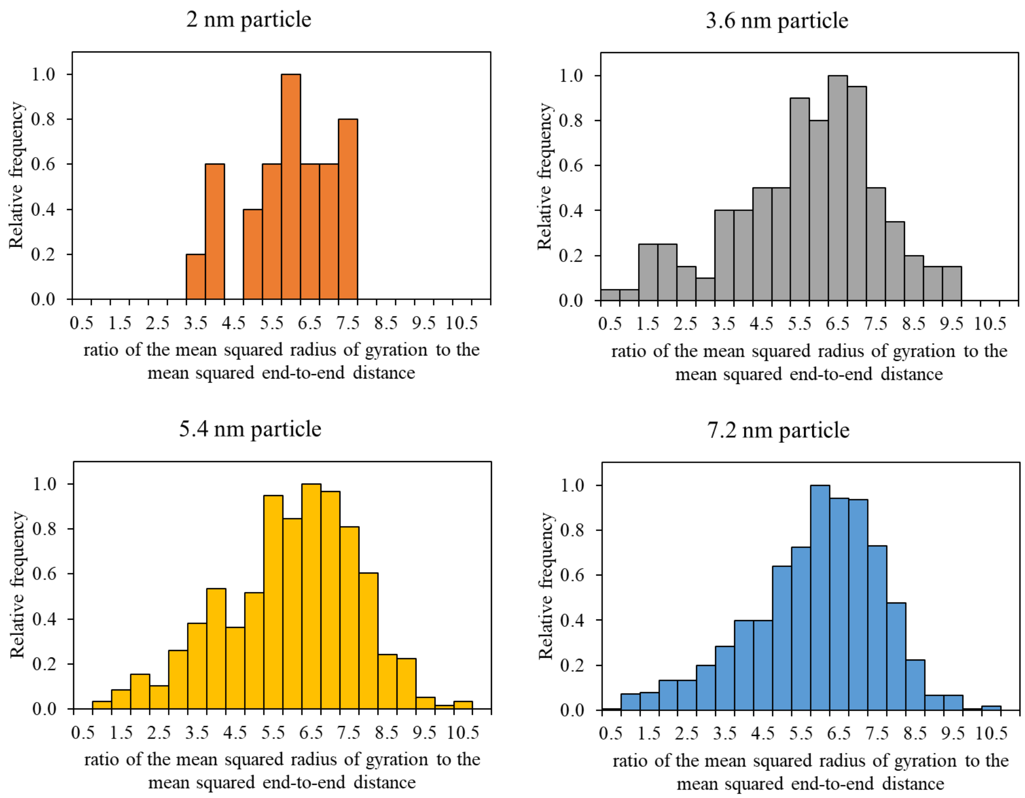

Figure S15. Distribution of polymer chain structure ratio (mean squared end-to-end distance to the mean squared radius of gyration) as a function of particle size (nm) in 10 wt% filler composition.

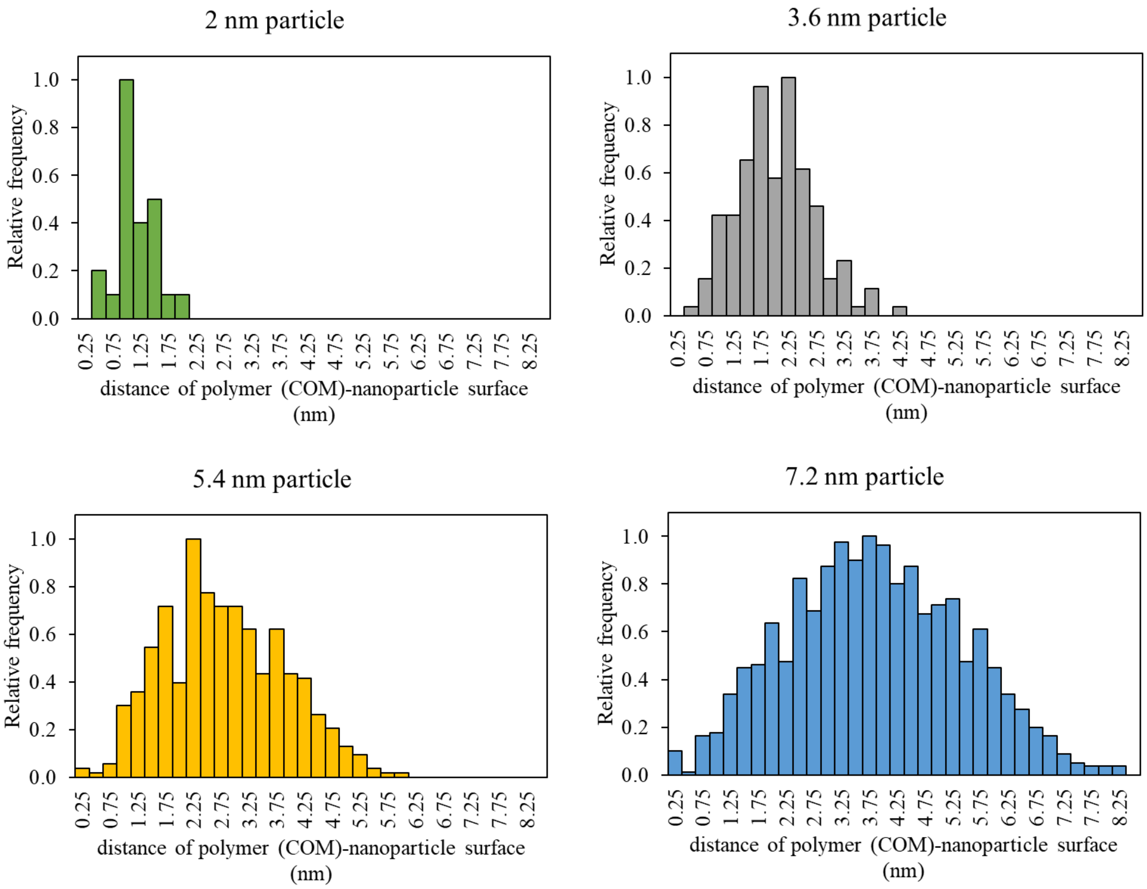

Figure S16. Distribution of distance of polymer COM to particle surfaces as a function of particle size (nm) in 10wt% filler composition.

References

1. Stote, R.H. and M. Karplus, *Zinc binding in proteins and solution: a simple but accurate nonbonded representation.* Proteins, 1995. **23**(1): p. 12-31.
2. Daglar, H. and S. Keskin, *Recent advances, opportunities, and challenges in high-throughput computational screening of MOFs for gas separations.* Coordination Chemistry Reviews, 2020. **422**.
3. Dubbeldam, D., et al., *RASPA: molecular simulation software for adsorption and diffusion in flexible nanoporous materials.* Molecular Simulation, 2016. **42**(2): p. 81-101.
4. Krokidas, P., et al., *Molecular Simulation Studies of the Diffusion of Methane, Ethane, Propane, and Propylene in ZIF-8.* Journal of Physical Chemistry C, 2015. **119**(48): p. 27028-27037.
5. Abraham, M.J., et al., *GROMACS: High performance molecular simulations through multi-level parallelism from laptops to supercomputers.* SoftwareX, 2015. **1-2**: p. 19-25.
6. Su, Z., et al., *Compression-Induced Deformation of Individual Metal–Organic Framework Microcrystals.* Journal of the American Chemical Society, 2015. **137**(5): p. 1750-1753.
7. Chapman, K.W., G.J. Halder, and P.J. Chupas, *Pressure-induced amorphization and porosity modification in a metal-organic framework.* J Am Chem Soc, 2009. **131**(48): p. 17546-7.
8. Macrae, C.F., et al., *Mercury 4.0: from visualization to analysis, design and prediction.* Journal of Applied Crystallography, 2020. **53**(1): p. 226-235.
9. Ghalei, B., et al., *Enhanced selectivity in mixed matrix membranes for CO2 capture through efficient dispersion of amine-functionalized MOF nanoparticles.* Nature Energy, 2017. **2**(7): p. 17086.
